# Engineered Spider Silk Proteins for Biomimetic Spinning of Fibers with Toughness Equal to Dragline Silks

**DOI:** 10.1002/adfm.202200986

**Published:** 2022-03-25

**Authors:** Tina Arndt, Gabriele Greco, Benjamin Schmuck, Jessica Bunz, Olga Shilkova, Juanita Francis, Nicola M Pugno, Kristaps Jaudzems, Andreas Barth, Jan Johansson, Anna Rising

**Affiliations:** ^1^ Department of Biosciences and Nutrition Karolinska Institutet Neo Huddinge 14183 Sweden; ^2^ Laboratory for Bioinspired, Bionic, Nano, Meta, Materials & Mechanics Department of Civil, Environmental and Mechanical Engineering University of Trento Via Mesiano 77 Trento 38123 Italy; ^3^ Department of Anatomy Physiology and Biochemistry Swedish University of Agricultural Sciences Uppsala 75007 Sweden; ^4^ School of Engineering and Materials Sciences Queen Mary University of London Mile End Road London E1 4NS UK; ^5^ Department of Physical Organic Chemistry Latvian Institute of Organic Synthesis Riga LV‐1006 Latvia; ^6^ Department of Biochemistry and Biophysics The Arrhenius Laboratories for Natural Sciences Stockholm University Stockholm 10691 Sweden; ^7^ Present address: Spiber Technologies AB AlbaNova University Center SE‐10691 Stockholm Sweden

**Keywords:** biomimetic materials, biomimetic spider silk fibers, fibers, protein engineering, recombinant protein production

## Abstract

Spider silk is the toughest fiber found in nature, and bulk production of artificial spider silk that matches its mechanical properties remains elusive. Development of miniature spider silk proteins (mini‐spidroins) has made large‐scale fiber production economically feasible, but the fibers’ mechanical properties are inferior to native silk. The spider silk fiber's tensile strength is conferred by poly‐alanine stretches that are zipped together by tight side chain packing in β‐sheet crystals. Spidroins are secreted so they must be void of long stretches of hydrophobic residues, since such segments get inserted into the endoplasmic reticulum membrane. At the same time, hydrophobic residues have high β‐strand propensity and can mediate tight inter‐β‐sheet interactions, features that are attractive for generation of strong artificial silks. Protein production in prokaryotes can circumvent biological laws that spiders, being eukaryotic organisms, must obey, and the authors thus design mini‐spidroins that are predicted to more avidly form stronger β‐sheets than the wildtype protein. Biomimetic spinning of the engineered mini‐spidroins indeed results in fibers with increased tensile strength and two fiber types display toughness equal to native dragline silks. Bioreactor expression and purification result in a protein yield of ≈9 g L^−1^ which is in line with requirements for economically feasible bulk scale production.

## Graphical abstract

Basic biological insights and rational protein engineering is used to design engineered spider silk proteins which are spun into fibers in a biomimetic spinning set‐up. The fibers show increased tensile strength compared to the original nonengineered fiber, and two of the fiber types display a toughness equal to native dragline silks. The high protein yields allow translation into industrial production.

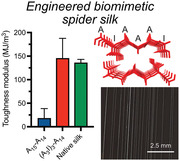

## 1 Introduction

Spider silk is nature's high‐performance fiber. Its unique combination of high tensile strength and extensibility results in an unsurpassed toughness which makes it very attractive for many industrial applications.^[^ ^1^, ^2^, ^3^ ^]^ Due to limited availability of the natural material, large scale production must involve the expression of the silk proteins (spidroins) in heterologous hosts.^[^ ^4^ ^]^

Spiders have up to seven different types of silk glands in which the spidroins are being produced, stored, and processed.^[^ ^5^ ^]^ The major ampullate gland makes the strongest silk, which is used in the dragline and for making the framework of the web.^[^ ^6^, ^7^, ^8^, ^9^, ^10^, ^11^ ^]^ The spidroins are synthesized by epithelial cells lining the major ampullate gland and are stored in the gland lumen as a highly concentrated dope.^[^ ^9^, ^12^, ^13^ ^]^ Changes in the microenvironment along the gland,^[^ ^14^ ^]^ for example, ion exchange, drop in pH from 8.0 to at least 5.7,^[^ ^15^ ^]^ increased shear forces,^[^ ^16^ ^]^ and dehydration^[^ ^7^ ^]^ lead to conformational transitions of the spidroins and fiber formation.^[^ ^15^, ^17^, ^18^, ^19^, ^20^ ^]^

Spidroins are composed of an N‐terminal domain (NT),^[^ ^21^ ^]^ a repetitive region that often is extensive^[^ ^22^ ^]^ and a C‐terminal domain (CT).^[^ ^18^ ^]^ The terminal domains are crucial for solubility of the spidroins during storage and regulate the assembly of the spidroins into a solid fiber.^[^ ^17^, ^18^, ^19^, ^20^, ^23^ ^]^ The repetitive region of most major ampullate spidroins (MaSps) contain up to 100 tandem repeats of poly‐Ala blocks and Gly‐rich motifs.^[^ ^22^, ^24^ ^]^ In the soluble dope, the spidroins are mostly in random coil and helical conformations,^[^ ^25^, ^26^, ^27^, ^28^, ^29^ ^]^ whereas the solid silk fiber contains nanosized crystals made up by stacked antiparallel β‐sheets embedded in amorphous structures.^[^ ^30^, ^31^, ^32^, ^33^, ^34^ ^]^ This heterogeneous structure of the silk fiber is important as the β‐sheet crystals confer the strength while the amorphous structures confer the extensibility to the fiber.^[^ ^10^, ^35^, ^36^ ^]^ The amorphous matrix, containing β‐turns and ordered structures with conformational similarities to collagen and poly‐proline helices, are dominated by the glycine‐rich regions. The β‐sheets, formed by the poly‐Ala blocks, orient with the β‐strands parallel to the fiber axis,^[^ ^37^, ^38^, ^39^, ^40^ ^]^ and the Ala side chain of a given β‐strand fill the space close to an α‐carbon in a neighboring β‐stand, analogous to a tightly packed steric zipper.^[^ ^41^, ^42^, ^43^ ^]^

There are two main strategies for producing artificial silk fibers; one being expression of insoluble spidroins with subsequent solubilization and fiber processing using organic solvents,^[^ ^44^, ^45^, ^46^, ^47^, ^48^, ^49^ ^]^ and another being a biomimetic approach involving only aqueous solutions throughout the purification and spinning procedures and in which the molecular mechanisms and triggers for fiber formation are replicated.^[^ ^50^, ^51^, ^52^, ^53^ ^]^ The first approach enables expression of large spidroins that can be spun into fibers with high tensile strength, but the protein yields are far from what is required for industrial production.^[^ ^54^, ^55^ ^]^ Using the second approach, mini‐spidroins composed of an NT, a short repeat region consisting of two poly‐Ala/Gly‐rich blocks and a CT, have been developed. Such mini‐spidroins are extremely water‐soluble and can be spun into fibers using biomimetic spinning set‐ups.^[^ ^51^, ^52^, ^53^, ^56^ ^]^ Moreover, one of these mini‐spidroins, NT2RepCT, can be produced at a yield of 14.5 g L^−1^ in bioreactor cultivations which vouch for economically feasible bulk production.^[^ ^55^, ^56^ ^]^ Fibers spun from NT2RepCT are superior compared to previously published as‐spun fibers, but still, the fibers only reach about 15% of the native silk fiber's tensile strength.^[^ ^1^, ^51^ ^]^ NMR spectroscopy revealed that the mini‐spidroin's two poly‐Ala blocks are in an α‐helical conformation in the soluble state and convert to β‐sheet conformation in the as‐spun wet fiber, as expected. However, upon drying the fiber, the poly‐Ala blocks are transitioning back to α‐helical conformation,^[^ ^57^ ^]^ which could lead to the inferior mechanical properties of dried NT2RepCT fibers compared to the native silk fiber. We therefore hypothesize that the mechanical properties of recombinant fibers could be improved by increasing the β‐strand propensity and inter‐β‐sheet interactions of the poly‐Ala blocks,^[^ ^58^ ^]^ as it has been suggested by replacing the poly‐alanines with amyloidogenic sequences.^[^ ^59^ ^]^

**Figure 1:**
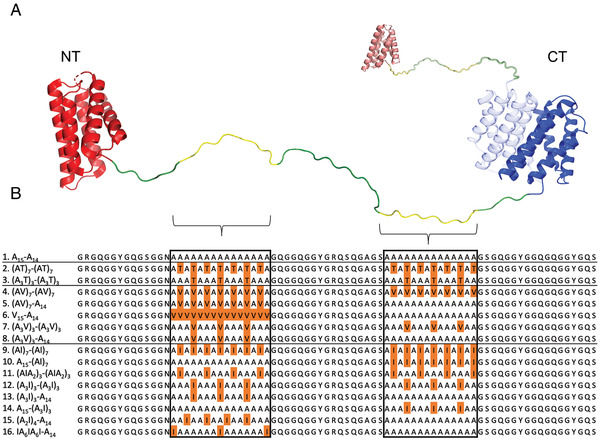
Schematic representation of the designed constructs. A) NT2RepCT (A_15_‐A_14_) is composed of an N‐terminal domain (NT, red; PDB: 4FBS), a repeat region with two poly‐Ala blocks (green and yellow), and a C‐terminal domain (CT, blue, PDB 3LR2). Both subunits of the soluble NT2RepCT dimer are shown (one is shaded). B) Protein sequence alignment of the repetitive region from A_15_‐A_14_ and engineered constructs thereof. Note that all constructs contain NT, a repeat part, and CT. Substitutions in the poly‐Ala blocks are indicated in orange.

Notably, Ala residues have a low propensity to form β‐strands, whereas more hydrophobic residues like Val, Cys, Ile, and Phe show a higher β‐strand propensity,^[^ ^60^ ^]^ and thus could be considered better candidates for forming stable β‐sheets in the silk fiber. However, being secretory proteins, the spidroins need to pass through the translocon when produced by the gland epithelium.^[^ ^61^ ^]^ If the nascent polypeptide chain contains segments that are rich in Val, Ile, Cys, or Phe the translocon will mediate insertion into the endoplasmic reticulum membrane,^[^ ^62^, ^63^ ^]^ and thus any spidroin segment rich in these amino acid residues would be trapped in the cell. In fact, Ala is the most hydrophobic residue that allows passage through the translocon, which suggests that the spidroins have evolved to optimize hydrophobicity in their β‐sheet forming segments to the extent possible for a secretory protein.^[^ ^58^, ^60^ ^]^ Intracellular expression in prokaryotes will bypass the restrictions imposed by the secretory pathway that native spidroins must adhere to since translation and accumulation of the target protein takes place in the cytosol. These fundamental biological principles led us to use rational design and protein engineering to generate mini‐spidroins that potentially can be produced at high yields in prokaryotic hosts and be used to generate stronger biomimetic artificial spider silk fibers (**Figure** **1**A,B). The Zipper database^[^ ^64^ ^]^ was used to screen a large panel of mini‐spidroins with designed modifications of the poly‐Ala blocks and candidates with low Rosetta energies were chosen for heterologous expression. Soluble target proteins were identified, characterized biochemically, and spun into fibers using a biomimetic spinning device. The mechanical performance of the fibers reveals that engineering of the repeat domain of mini‐spidroins is possible and can result in fibers with increased tensile strength.

## 2 Results and Discussion

Based on the β‐strand/α‐helix propensity ratios of amino acid residues as well as their hydrophobicity, Ile and Val were chosen to design 13 different constructs with substitutions in the poly‐Ala blocks of the original NT2RepCT sequence (referred to as A_15_‐A_14_ to reflect the composition of the two poly‐Ala blocks), (Figure 1). Additionally, the less hydrophobic residue Thr was used since it is branched at the β‐carbon and hence favors β‐strand conformation.^[^ ^65^, ^66^ ^]^

Figure 1B shows the amino acid sequences of the repetitive regions from A_15_‐A_14_ and engineered constructs with substitutions indicated (complete sequences can be found in Table S1, Supporting Information). Substitutions were mainly introduced at every second position resulting in β‐strands with mutated side chains on the same side. Mutations were introduced in either both (e.g., (AV)_7_‐(AV)_7_) or only in one of the poly‐Ala blocks (e.g. (AV)_7_‐A_14_). The number of substitutions varied between 15 (e.g., V_15_‐A_14_, in which all Ala are replaced by Val in the first poly‐Ala block) and 3 as in, for example, (A_3_V)_3_‐(A_14_), which contains Val substitution at every fourth position in the first poly‐Ala block. A few additional constructs were designed to analyze the impact of the position of the substituted residues, for example, (A_3_I)_3_‐A_14_, A_15_‐(A_3_I)_3_ and IA_6_IA_6_I‐A_14_ that all have three Ile substitutions but in different locations.

The packing of β‐sheets in amyloid‐like fibrils involve steric zippers,^[^ ^41^, ^67^ ^]^ which are also found in spider silk β‐sheet crystals.^[^ ^36^, ^43^ ^]^ Steric zippers are formed by tightly bound β‐strands with high complementarity of the involved side chains.^[^ ^41^, ^67^ ^]^ The Zipper database predicts the stability and propensity of hexapeptides in a given amino acid sequence to form steric zippers by calculating the energies of the interstrand interactions. Rosetta energies equal or below −23 kcal mol^−1^ suggest a high propensity to form steric zippers.^[^ ^64^ ^]^

**Figure 2:**
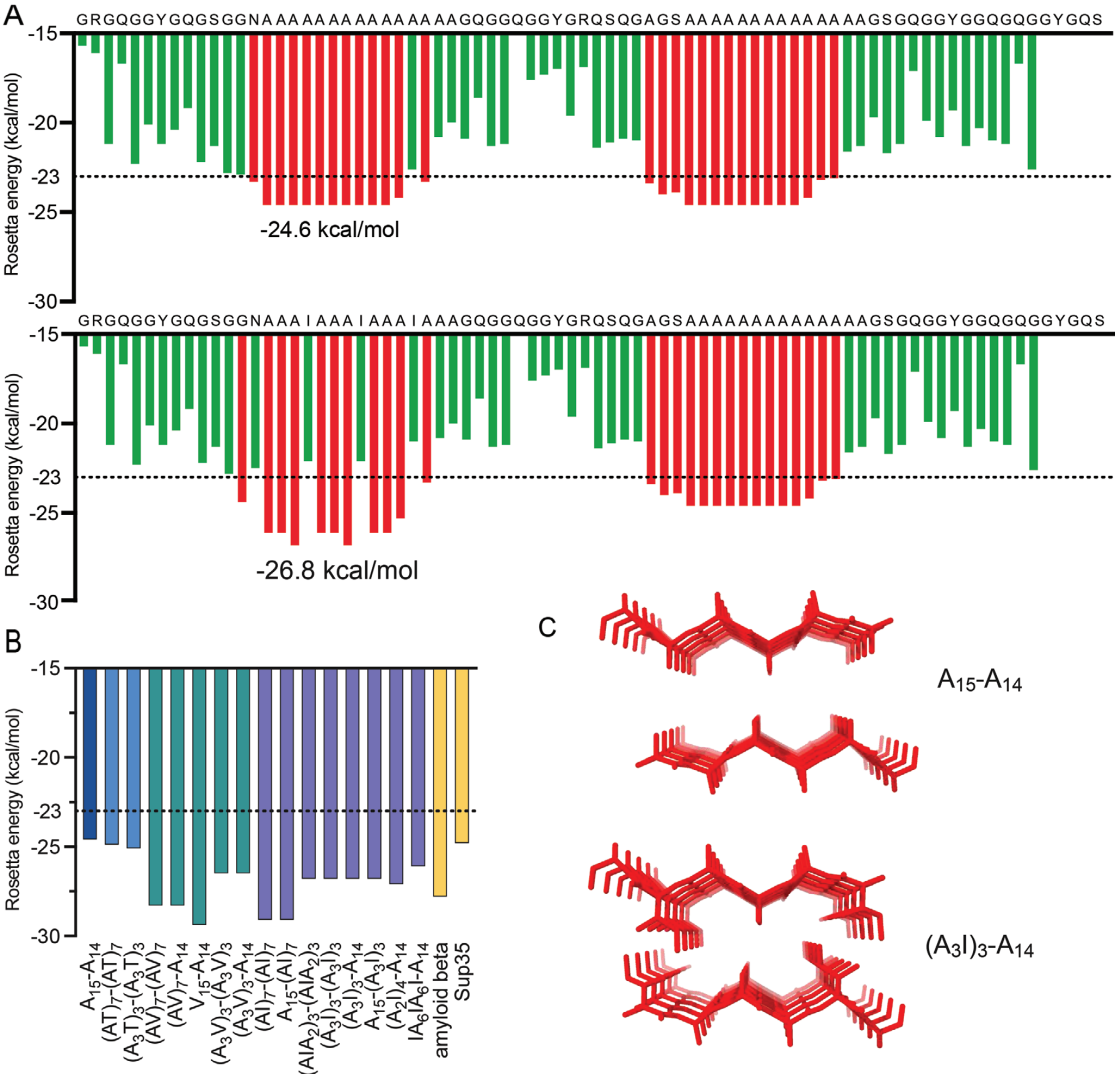
Rosetta energy profiles of A) A_15_‐A_14_ and (A_3_I)_3_‐A_14_ (profiles for all designed proteins are found in Figure S1 and Table S2, Supporting Information). Bars show Rosetta energies for moving hexapeptides (indicated at the first residue of each hexapeptide), red bars indicate Rosetta energies equal or below −23 kcal mol^−1^ (dashed line). Green bars indicate Rosetta energies above the threshold and are unlikely to form steric zippers (https://services.mbi.ucla.edu/zipperdb/).^[^ ^64^ ^]^ B) Bars indicate the Rosetta energy of the hexapeptide with the lowest predicted energy from A_15_‐A_14_ and the engineered mini‐spidroins (all hexapeptides are shown in Table S2, Supporting Information). C) Hypothetical zipper structure of two β‐sheets composed of hexapeptides AAAAAA from A_15_‐A_14_ and AIAAI derived from (A_3_I)_3_‐A_14_, respectively.

**Figure** **2**A shows the Rosetta energies estimated for constructs A_15_‐A_14_ and (A_3_I)_3_‐A_14_ (corresponding profiles for all engineered mini‐spidroins are shown in Figure S1, Supporting Information, and summarized in Table S2, Supporting Information, and Figure 2B). As expected, the hexapeptides in the poly‐Ala region of the A_15_‐A_14_ construct have low Rosetta energies (−24.6 kcal mol^−1^) and thus should be able to form steric zippers (Figure 2C). All designed constructs contain at least one hexapeptide with a Rosetta energy lower than that of A_15_‐A_14_ (Table S2, Supporting Information), ranging from −24.9 to −29.4 kcal mol^−1^ (for (AT)_7_‐(AT)_7_ and V_15_‐A_14_, respectively). Generally, the effect on the Rosetta energies increased with an increasing number of hydrophobic replacements in the poly‐Ala region.

Of the 15 designed proteins, seven were overexpressed and six were highly overexpressed in *E. coli* BL21 cells (**Table** **1** and Figures S2 and S3, Supporting Information). Constructs with Val substitutions had lower expression levels than corresponding constructs with Ile substitutions, but the number of substitution and the hydrophobicity did not have any general impact on expression levels (Figure S4, Supporting Information). The (AT)_7_‐(AT)_7_ construct did not express well which could be due to that this repeat was designed to resemble a “CAT tail” which is known to lead to aggregation of the nascent polypeptide chain and to degradation by the proteasome.^[^ ^68^ ^]^

**Table 1:** Summary of number of substitutions, expression levels, solubility after cell lysis, protein yield, and spinnability into fibers of the engineered proteins. Expression levels, solubility after cell lysis, and spinnability into fibers are rated from very high (+++), intermediate (++), low (+), and not at all (0). Rating of expression level and solubility after cell lysis were estimated by appearance of the target band on SDS‐PAGE (Figures S2 and S3, Supporting Information). (−) indicates not tested. (^1^) indicates degradation during expression. (*) marks purification using gravity columns instead of FPLC

Construct	Number of substitutions	Expression levels	Solubility after cell lysis	Average protein yield [mg L^−1^ culture]	Spinnability into fibers
1. A_15_‐A_14_	0	+++	+++	250	+++
2. (AT)_7_‐(AT)_7_	14	+	−	−	−
3. (A_3_T)_3_‐(A_3_T)_3_	6	++	+++	58*	+++
4. (AV)_7_‐(AV)_7_	14	+++	0	−	−
5. (AV)_7_‐A_14_	7	+++	0	−	−
6. V_15_‐A_14_	15	+/++^1^	0	−	−
7. (A_3_V)_3_‐(A_3_V)_3_	6	+++	+++	139*	+++
8. (A_3_V)_3_‐A_14_	3	++	+++	216	+++
9. (AI)_7_‐(AI)_7_	14	+	+	4*	−
10. A_15_‐(AI)_7_	7	+	+	−	−
11. (AIA_2_)_3_‐(AIA_2_)_3_	8	++	+	−	−
12. (A_3_I)_3_‐(A_3_I)_3_	6	+++	+++	94*	+
13. (A_3_I)_3_‐A_14_	3	+++	+++	207	+++
14. A_15_‐(A_3_I)_3_	3	+++	+++	233	+++
15. (A_2_I)_4_‐A_14_	4	++	+++	243	+++
16. IA_6_IA_6_I‐A_14_	3	++	++	139	−

In addition to A_15_‐A_14_, seven of the constructs were found mainly in the soluble fraction after cell lysis in 20 mm Tris‐HCl, and four constructs were in both the soluble and insoluble fraction (Table 1 and Figure S3, Supporting Information). Increased hydrophobicity, number of substitutions, and lower Rosetta energies correlated with lower solubility after cell lysis (Figure S4, Supporting Information). 9 of the 15 designed constructs plus the control A_15_‐A_14_ yielded sufficient soluble protein for purification. Nondenaturing immobilized metal affinity chromatography yielded between 4 and 243 mg of pure target protein per 1 L shake flask culture (average of 10 × 1 L cultures). Notably, six of the engineered mini‐spidroins gave very high yields (>100 mg L^−1^ Table 1). (AV)_7_‐(AV)_7_, (AV)_7_‐A_14_, and V_15_‐A_14_ expressed well but were insoluble after lysis, likely due to high hydrophobicity of the engineered segments. Expression and purification of the A_15_‐(AI)_7_ and (AIA_2_)_3_‐(AIA_2_)_3_ constructs did not result in enough soluble protein for further characterization. The constructs that showed intermediate to high expression levels but were insoluble after cell lysis were treated with 8 m urea but could not be solubilized to the extent needed for enabling purification of enough protein for fiber spinning (not shown).

The position of the Ile replacements within one Ala block had an impact on the protein yield but whether these were located in the first or second poly‐Ala block did not matter. For example, (A_3_I)_3_‐A_14_ and A_15_‐(A_3_I)_3_ both have three Ile substitutions in the first and second poly‐Ala block, respectively, and show comparable yields. In contrast, (A_3_I)_3_‐A_14_ and IA_6_IA_6_I‐A_14_ have the same number of Ile replacements in the first block, but their location differ as does the yield (207 vs 139 mg L^−1^ culture for (A_3_I)_3_‐A_14_ and IA_6_IA_6_I‐A_14_, respectively).

**Figure 3:**
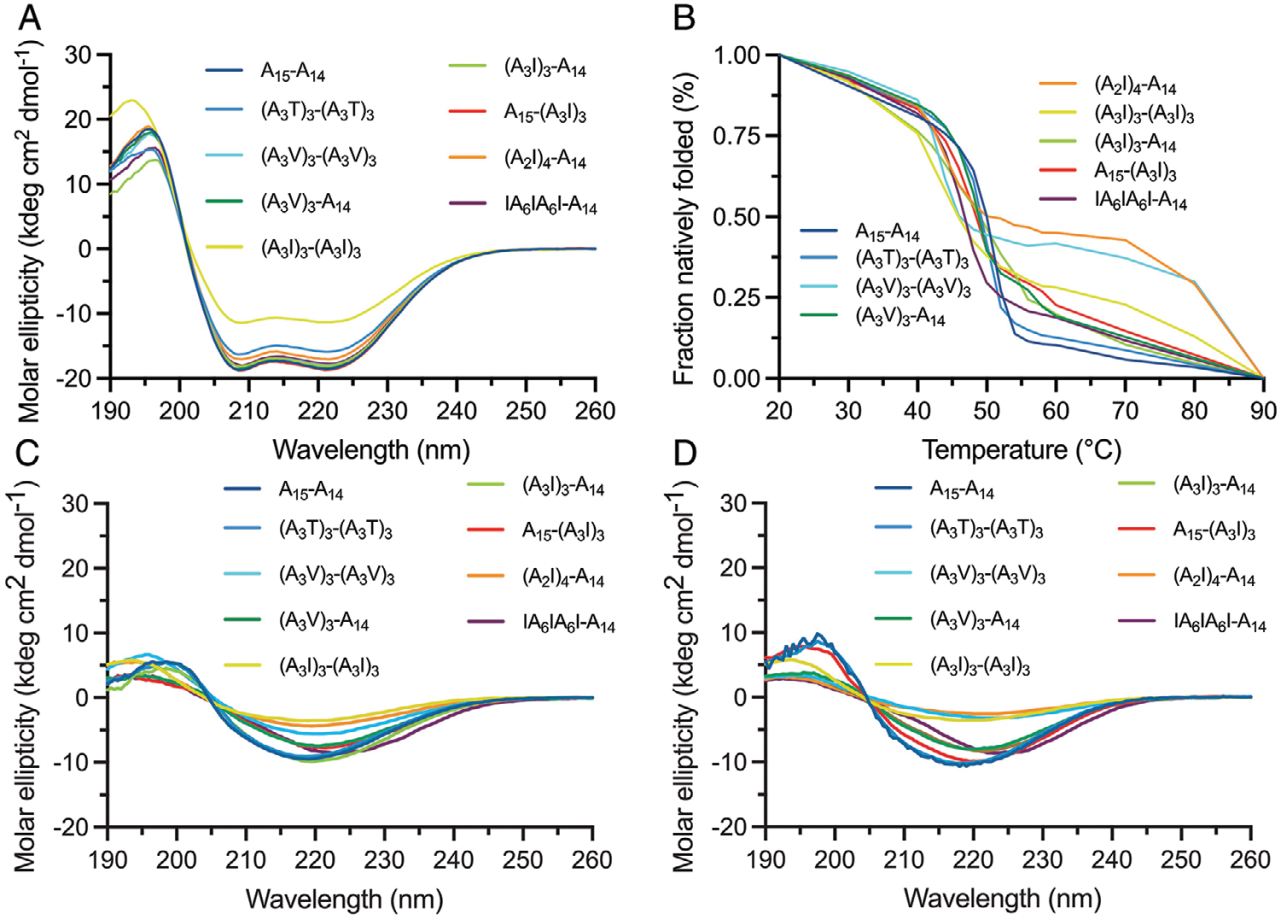
CD spectroscopy of purified engineered mini‐spidroins. A) Initial spectra at 20 °C and B) molar ellipticity measured at 222 nm from 20 to 90 °C was converted to fraction natively folded (%) and then normalized. CD spectroscopy of different constructs C) heated to 90 °C and D) after cooling to 20 °C.

Next, we investigated the secondary structure content and the thermal stability of the purified constructs by circular dichroism (CD) spectroscopy (**Figure** **3**). We found that all constructs had an overall α‐helical secondary structure (Figure 3A) which indicates that the amino acid substitutions did not affect the secondary structure of the soluble proteins to any large extent. Heating to 90 °C led to a decreased signal for all constructs and concomitant transition to β‐sheet dominated secondary structures (Figure 3C). The heat‐induced conformational changes were irreversible upon cooling of the samples (Figure 3D). ﻿ Melting curves for all constructs showed that the proteins unfolded around 46–50 °C, which is in line with reports on the isolated terminal domains,^[^ ^15^ ^]^ and means that the substitutions in the repetitive region of the mini‐spidroins only had a minor effect on the thermal stability of the proteins (Figure 3B).

**Figure 4:**
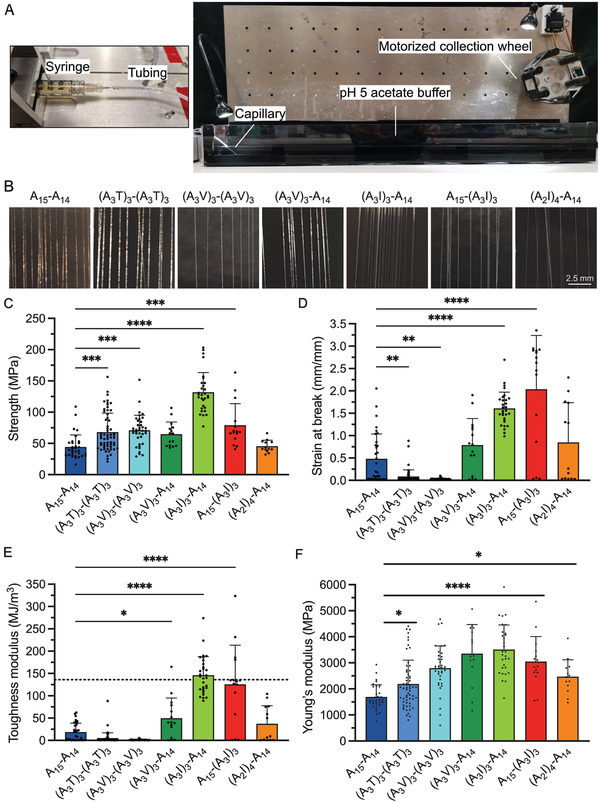
Mechanical properties of spinnable engineered mini‐spidroins in comparison with A_15_‐A_14_. A) Photographs of the biomimetic spinning set‐up; a video of the spinning can be found in Video S1, Supporting Information. B) Photographs of spun fibers. C) Strength, D) strain at break, E) toughness modulus, dashed line indicates toughness modulus of a native dragline silk,^[^ ^10^ ^]^ and F) Young's modulus. Whiskers show standard deviation. *p < 0.05; **p < 0.01; ***p < 0.001; ****p < 0.0001. Representative stress–strain graphs for all spinnable engineered mini‐spidroins are shown in Figure S5A–G, Supporting Information. The diameters of the fibers are shown in Figure S5H, Supporting Information. The values and corresponding standard deviations are shown in Table S3, Supporting Information.

Out of the nine engineered mini‐spidroins that were successfully purified (excluding A_15_‐A_14_), eight could be concentrated to at least 200 mg mL^−1^ to generate spinning dopes, while (AI)_7_‐(AI)_7_ yielded too little protein (Table 1). The dopes made from the eight constructs were transferred to syringes and extruded through a thin glass capillary into a low pH aqueous buffer according to a previously described biomimetic spinning procedure.^[^ ^50^, ^51^ ^]^ Seven engineered mini‐spidroins could be spun into fibers, and only the IA_6_IA_6_I‐A_14_ protein aggregated prematurely in the syringe. One of the mini‐spidroins, (A_3_I)_3_‐(A_3_I)_3_, formed fibers that were too fragile to be retrieved. The reason for the poor integrity of the (A_3_I)_3_‐(A_3_I)_3_ fibers is not known but was not related to premature aggregation in the dope. The other six engineered fiber types, plus the A_15_‐A_14_ fibers, were successfully collected onto a motorized wheel at the end of the spinning bath (**Figure** **4**A and Video S1, Supporting Information). There was no difference in the appearance of the spun fibers (Figure 4B) and the diameter of the different fiber types, determined by light microscopy, varied between 4 and 19 µm (Figure S5H and Table S3, Supporting Information). The reason for the differences in diameter between the different fiber types is not known but is likely linked to differences in the properties of the proteins since the spinning conditions were kept constant.

The tensile strength of all fibers spun from engineered proteins increased significantly compared to A_15_‐A_14_ except for (A_3_V)_3_‐A_14_ and (A_2_I)_4_‐A_14_ (Figure 4 and Table S3, Supporting Information). The two similar fiber types (A_3_I)_3_‐A_14_ and A_15_‐(A_3_I)_3_ displayed the highest increase in strength, the former reaching 131 MPa, which is almost three times higher than that of A_15_‐A_14_ (Figure 4C). This indicates that rational protein engineering of the spidroin poly‐Ala blocks indeed can result in increased fiber tensile strength and stiffness. Unexpectedly, the introduced amino acid substitutions also had a high impact on the extensibility of the fiber, as the strain at break varied from 0.03 to 2.0 mm mm^−1^ (Figure 4D and Table S3, Supporting Information). The two strongest fiber types ((A_3_I)_3_‐A_14_ and A_15_‐(A_3_I)_3_) displayed an exceptional increase in strain (to 1.6 and 2.0 mm mm^−1^, respectively), while (A_3_V)_3_‐A_14_, (A_2_I)_4_‐A_14_ fibers showed moderately increased strain (0.79 and 0.85 mm mm^−1^, respectively) compared to A_15_‐A_14_ (0.45 mm/mm). (A_3_T)_3_‐(A_3_T)_3_ and (A_3_V)_3_‐(A_3_V)_3_ fibers were the least extensible (0.03 and 0.08 mm mm^−1^, respectively). These two proteins contain substitutions in both poly‐Ala blocks and, possibly, the reason for the inferior strain of these fibers could be an increased propensity of the engineered segments to interact intra‐molecularly over forming intermolecular contacts.

Apparently, the mechanical properties of artificial spider silk fibers can be significantly improved by introducing Ile in every fourth position in the first or second poly‐Ala block. These two mini‐spidroins, (A_3_I)_3_‐A_14_ and A_15_‐(A_3_I)_3_, formed fibers with a toughness modulus that is comparable to native dragline silk (146 and 125 MJ m^−3^, respectively, compared to 136 MJ m^−3^ for a native dragline silk from *Argiope argentata*), (Figure 4E).^[^ ^10^ ^]^ Fibers formed by (A_3_V)_3_‐A_14_ and (A_2_I)_4_‐A_14_ also reached a significantly higher toughness modulus than A_15_‐A_14_ (50 and 37 MJ m^−3^, respectively, compared to 18 MJ m^−3^).

**Figure 5:**
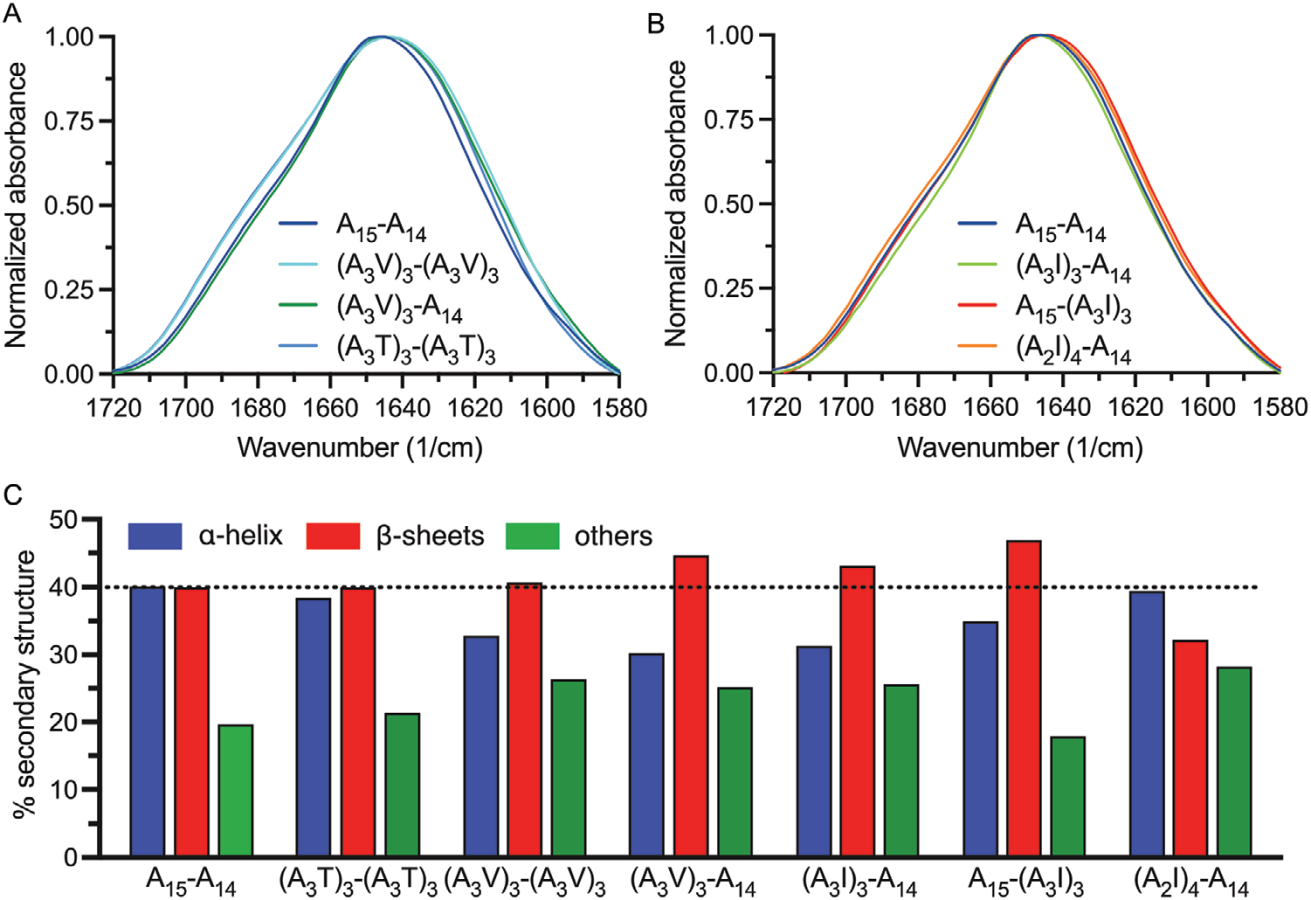
FTIR spectroscopy of engineered fibers. Normalized and baseline‐subtracted absorbance spectrum in the amide I region of A) A_15_‐A_14_, (A_3_V)_3_‐(A_3_V)_3_, (A_3_V)_3_‐A_14_, and (A_3_T)_3_‐(A_3_T)_3_ and B) A_15_‐A_14_, (A_3_I)_3_‐A_14_, A_15_‐(A_3_I)_3_, and (A_2_I)_4_‐A_14_. C) Percent secondary structure content determined by cofitting the absorbance spectrum and the second derivative. Horizontal line indicates β‐sheet content of A_15_‐A_14_. Fits of absorbance spectra and second derivative of fibers spun are shown in Figure S7, Supporting Information.

**Figure 6:**
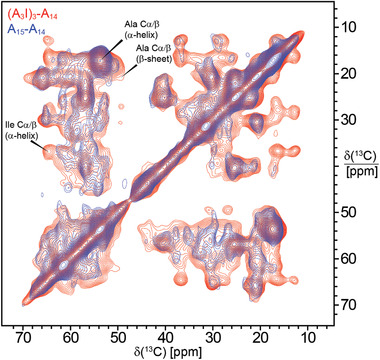
Solid‐state NMR ^13^C‐^13^C correlation spectra (aliphatic region) of A_15_‐A_14_ (blue) and (A_3_I)_3_‐A_14_ (red) fibers. The Cα/Cβ correlations of Ala and Ile in α‐helical and β‐sheet conformation are indicated.

To investigate the link between fiber secondary structure content and mechanical properties, we used attenuated total reflection Fourier‐transform infrared (ATR‐FTIR) spectroscopy. The results, shown in **Figure** **5** and Figure S7 and Table S4, Supporting Information, indicate that no large differences in secondary structure content between fibers were detected, but (A_3_V)_3_‐A_14_, (A_3_I)_3_‐A_14_ and A_15_‐(A_3_I)_3_ had a slightly increased β‐sheet content, along with decreased α‐helix/random coil content compared to A_15_‐A_14_ fibers. However, the (A_3_V)_3_‐(A_3_V)_3_ and (A_2_I)_4_‐A_14_ fibers failed to show increased β‐sheet content compared to A_15_‐A_14_ fibers and we could detect no strong correlations between secondary structure content and mechanical properties of the fiber (Figure S6, Supporting Information). Thus, ATR‐FTIR spectroscopy of the different fiber types did not detect any significant differences in secondary structure content. Therefore, we decided also to use solid‐state NMR spectroscopy to investigate the unmodified fibers (A_15_‐A_14_) and the best performing engineered fibers, (A_3_I)_3_‐A_14_. As expected, more Ala residues were found in a β‐sheet conformation in (A_3_I)_3_‐A_14_ compared to A_15_‐A_14_ fibers (**Figure** **6**).

The altered mechanical properties of the fibers made from the engineered spidroins indicate that intermolecular interactions in the spidroins are affected. In the native dragline silk fiber, pulling the fiber first results in reversible deformation of the amorphous regions up until the yielding point, after which the hydrogen bonds in the amorphous region break, resulting in softening of the material.^[^ ^36^, ^43^ ^]^ When the amorphous protein chains are extended, the load is transferred onto the β‐sheet crystals leading to a stiffening of the fiber. Upon further increased load, the β‐sheet crystals undergo stick‐slip deformation and the fiber breaks.^[^ ^36^, ^43^, ^69^ ^]^ The increased tensile strength of the fibers made from engineered proteins suggests that our strategy to increase the β‐strand propensity and inter‐β‐sheet interactions indeed can result in stronger fibers, although some of the engineered fibers concomitantly displayed a decreased strain. Theoretically, increased β‐sheet formation and intermolecular interactions in the stacked β‐sheets could not only result in increased fiber strength, but also increased extensibility, since the amorphous region would be allowed to extend fully before the load is transferred to the crystalline region. In lack of poly‐Ala β‐sheet crystals, as in the A_15_‐A_14_ fibers, the intermolecular contacts may be too weak to allow a full extension of the amorphous protein chains before fiber failure. At the same time, it may be disadvantageous that all β‐sheets stack in crystals since only about 40% of the Ala residues in the native dragline silk are found in this conformation and the rest form less ordered β‐sheets.^[^ ^70^ ^]^ In this study, introducing replacements in both poly‐Ala blocks resulted in fibers with dramatically reduced strain which suggest a suboptimal packing of the proteins in the fiber.

Since the (A_3_I)_3_‐A_14_ fibers displayed superior mechanical properties, these fibers are attractive candidates for bulk‐scale production. Previously, A_15_‐A_14_ has been shown to express at very high levels (≈21 g L^−1^) in a bioreactor‐based *E. coli* fed‐batch culture.^[^ ^56^ ^]^ Following the same protocol, the expression level of (A_3_I)_3_‐A_14_ amounted to 13 g L^−1^ and the final yield after purification using an automated purification protocol was 8.9 g L^−1^ (Figure S8A,B, Supporting Information). To our knowledge, these yields are the second highest reported for any recombinant spidroin produced in *E. coli* and line with what is required for economically viable bulk production.^[^ ^55^, ^56^ ^]^ After purification, (A_3_I)_3_‐A_14_ was concentrated to 300 mg mL^−1^ and could easily be spun into fibers. Notably, 8.9 g recombinant silk protein is enough to produce an ≈18 km long fiber. When comparing (A_3_I)_3_‐A_14_ fibers produced from proteins recovered from bioreactor and shake flask fermentations, respectively, the former had slightly lower strength (Figure S9D, Supporting Information). However, the bioreactor produced (A_3_I)_3_‐A_14_ fibers still had a significantly higher tensile strength and strain compared to A_15_‐A_14_ fibers (Figure S9, Supporting Information).

## 3 Conclusion

Using biological principles, we employed protein engineering to design mini‐spidroins with predicted increased β‐sheet propensities and increased inter‐β‐sheet binding strengths. Prokaryotic expression, protein purification, and biomimetic fiber spinning resulted in four different types of fibers with significantly improved tensile strength compared to the original mini‐spidroin. Using this strategy, we successfully produced the first biomimetic fibers with toughness values matching those of native dragline silk fibers. Finally, we show that these fibers can be produced at very high yields in bioreactors, vouching for feasible large‐scale production.

## 4 Experimental Section

### Designed Mini‐Spidroins

All expressed proteins were composed of a 6xHis‐tag, an NT from *Euprosthenops australis* MaSp1 and a CT from *Araneus ventricosus* minor ampullate spidroin (MiSp). Between NT and CT, a repetitive part was inserted containing two poly‐Ala and three glycine‐rich repeats from *E. australis* MaSp1 (NT2RepCT) as described previously.^[^ ^51^ ^]^ Engineered variants were designed that contained amino acid residue substitutions in the poly‐Ala blocks of the repetitive region as described in the results section. Note that the constructs were named after their substitutions in the poly‐Ala blocks but contained NT, CT, and the glycine‐rich regions as well, for example, NT2RepCT was referred to as A_15_‐A_14_. Amino acid sequences corresponding to the designed repeat regions were converted into gene sequences and codon optimized for expression in *E. coli* (Geneious), ordered from Eurofins Genomics, Germany, and subcloned between NT and CT (using EcoRI and BamHI restriction sites) of the existing NT2RepCT plasmid.^[^ ^51^ ^]^ See Table S1, Supporting Information, for full sequences.

### Fibrillation Propensity and Hydrophobicity

The Zipper database^[^ ^64^ ^]^ was used to estimate the fibrillation propensity and Rosetta energies of engineered constructs (only the repetitive region) as silk was proposed to form β‐sheets that pack into crystals.^[^ ^15^, ^32^, ^71^, ^72^ ^]^ The Zipper database calculated the Rosetta energy^[^ ^73^ ^]^ and evaluated self‐complementary binding of moving hexapeptides.^[^ ^41^, ^67^ ^]^ The Rosetta energy combined several free energy functions to model and analyze given protein structures, and energies equal or below −23 kcal mol^−1^ indicated high fibrillation propensity.^[^ ^64^ ^]^ Lower energies implied higher stability of two β‐strands in a zipper conformation. The hydrophobicity was calculated with https://web.expasy.org/protparam/.^[^ ^74^, ^75^, ^76^ ^]^

### Protein Expression Using Shake Flask Cultures

Protein expression was performed as described previously.^[^ ^50^ ^]^ In brief, the constructs were transformed in BL21 (DE3) *E. coli* cells and grown in Luria broth (Miller, VWR, USA) in shake flasks at 30 °C and 110 rpm containing kanamycin until the OD_600_ reached 0.9. To induce recombinant protein expression, 0.15 mm isopropyl β‐d‐1‐thiogalactopyranoside (final concentration; VWR, USA) was added and the temperature was lowered to 20 °C. Expression took place overnight after which the cells were harvested and stored at −20 °C.

### Protein Purification and Concentration

Cell lysis was done with a high‐pressure cell disrupter (T‐S Series Machine, Constant Systems Limited). Following centrifugation, the supernatant was purified by Ni‐immobilized metal affinity column (IMAC), (Äkta start, GE Healthcare, USA or manual). After loading the supernatant on a HisPrep FF 16/10 or manual packed column (GE Healthcare, USA), the column was washed with 4–5 column volumes (CV) of 20 mm Tris‐HCl followed by 4–5 CV of 2 mm imidazole in 20 mm Tris‐HCl, pH 8. The protein was eluted with 200 mm imidazole in 20 mm Tris‐HCl. After dialysis against 20 mm Tris‐HCl, pH 8, the protein was analyzed by SDS‐PAGE for quality control. Depending on the solubility of the construct, the proteins were concentrated to 200–400 mg mL^−1^ with centrifugal concentrators (Vivaspin 20, 10 kDa MWCO, GE Healthcare, USA) and then frozen at −20 °C until further use.

### CD Spectroscopy

Protein concentrations of 10 µm in 20 mm phosphate buffer were measured in a 300 µL cuvette with a 1 mm path length using a J‐1500 CD spectrometer (JASCO, USA). Temperature scans were performed between 20 and 90 °C at a heating rate of 1 °C min^−1^ and spectra were recorded from 260 to 190 nm. After heating, the samples were cooled to 20 °C for 15 min to observe reversibility of the conformational changes. The means of five scans per temperature were smoothed and converted to molar residue ellipticity. Thermal unfolding curves were plotted by taking the molar residual ellipticity at 222 nm and the fraction natively folded was converted with the formula (CD_measured_ − CD_end_)/(CD_start_ − CD_end_) and then normalized. After cooling precipitates were visible in the cuvette.

### Biomimetic Fiber Spinning

Artificial fiber spinning was performed similarly as described previously.^[^ ^50^ ^]^ Round‐glass capillaries (G1, Narishige, UK, inner diameter of 0.6 mm) were pulled with a Micro Electrode Puller (Stoelting co. 51217) to a diameter between 25 and 78 µm. A 1 mL syringe with Luer Lok tip (BD, USA) was filled with the concentrated proteins and connected to a 27 G steel needle (Braun, Germany). The needle was connected to the pulled‐glass capillary via polyethylene tubing. The protein was ejected at a flow rate of 17 µL min^−1^ (neMESYS low‐pressure syringe pump, Cetoni, Germany) into an 80 cm long bath containing spinning buffer (750 mm acetate buffer, 200 mm NaCl, pH 5.0) and rolled onto collection frames in air with minimal stretching of the fibers. Each construct was spun at least twice at different occasions.

### Mechanical Testing of the Fibers

Fibers were mounted with tape on paper frames with a square window (1 cm × 1 cm) and the diameter of the fibers was measured with an optical microscope (Nikon, Japan) at ten locations along each fiber and the average diameter was calculated. The frames were placed into a tensile tester (5943‐Instron, USA equipped with a 5N load cell), cut and the fiber was pulled at a strain‐rate of 6 mm min^−1^. All the tests were performed at relative humidity lower than 35% to not affect the mechanical properties of the silk.^[^ ^77^ ^]^ The types and number of fibers tested were: A_15_‐A_14_ *n* = 33, (A_3_I)_3_‐A_14_ *n* = 30, (A_3_T)_3_‐(A_3_T)_3_ *n* = 60, (A_3_V)_3_‐(A_3_V)_3_ *n* = 38, (A_3_V)_3_‐A_14_ *n* = 15, A_15_‐(A_3_I)_3_ *n* = 13, (A_2_I)_4_‐A_14_ *n* = 15. The engineering stress was calculated by dividing the measured force by the area of the cross section (calculated from the average diameter assuming a circular cross section). The engineering strain was calculated by dividing the displacement by the gauge length. Toughness modulus was obtained by calculating the area under the stress–strain curve and the Young's modulus was obtained from the slope at the initial linear elastic phase of the stress–strain curve.

### FTIR Spectroscopy

FTIR spectra of fiber bundles were recorded on a Vertex 70 instrument equipped with a diamond ATR unit (Platinum‐ATR, Bruker, Germany) and a mercury cadmium telluride‐detector (Bruker, Germany). The instrument was continuously purged with dried air and the spectra confirmed that water vapor correction was not necessary. 1000 scans with a resolution of 2 cm^−1^ were recorded. ﻿Before every sample spectrum measurement, a background spectrum without a sample was recorded and used to calculate the absorbance spectrum. For each sample, six spectra were taken by pressing fiber bundles on the ATR crystal with three fiber bundles oriented perpendicular to the beam and three fiber bundles parallel to it.

The “Kinetics” software, written by Erik Goormaghtigh (Université Libre de Bruxelles, Belgium) was used to process the spectra. The six spectra of each sample were averaged and the baseline was subtracted (﻿polynomial baseline with baseline points: 1740, 1730, 1580, and 1578 cm^−1^) from the amide I band (1705–1595 cm^−1^). The second derivative was calculated from the absorbance spectrum, smoothed with a 15‐point Savitzky–Golay algorithm and scaled to match the absorbance values (factor = 600). The absorbance and second derivative spectra were cofitted simultaneously to analyze the secondary structure content.^[^ ^78^ ^]^ Eight component bands were fitted (initial peak positions: 1695, 1680, 1669, 1651, 1633 1622, 1613, and 1599 cm^−1^) and ﻿bands were allowed to move ±5 cm^−1^ from that initial center peak position. Each component band was assigned to a secondary structure according to literature.^[^ ^79^, ^80^, ^81^, ^82^, ^83^ ^]^ The component band fitted at a center peak position of ≈1695 cm^−1^ was assigned to antiparallel β‐sheets. The component band fitted at ≈1651 cm^−1^ was assigned to α‐helix/random structures. Bands at ≈1633, ≈1622, and ≈1613 cm^−1^ were assigned to different types of β‐sheets according to a study of *B. mori* silk fibers:^[^ ^84^ ^]^ the 1633 cm^−1^ band likely corresponded to distorted or twisted beta‐sheets, while the ≈1622 and ≈1613 cm^−1^ bands were assigned to more planar sheets and had previously been proposed to differ in their methyl group orientations in *B. mori* silk fibers.^[^ ^84^, ^85^ ^]^ These assignments follow the known relationship between band position and planarity of β‐sheets.^[^ ^86^ ^]^ Bands at ≈1680 and ≈1669 cm^−1^ were assigned to other secondary structures and that at 1599 cm^−1^ was assigned to side chains^[^ ^87^ ^]^ as A_15_‐A_14_ (and all other constructs) contain 2.3% Glu and 1.4% Arg. The areas of the component bands were divided by the total fitted area of all bands assigned to amide I vibrations (excluding the side chain band) to calculate the relative secondary structure content.

### NMR Spectroscopy

The solid‐state NMR spectra of uniformly ^13^C, ^15^N‐labeled A_15_‐A_14_ and (A_3_I)_3_‐A_14_ fibers were recorded on an 800 MHz Bruker Avance III HD NMR spectrometer equipped with a 3.2 mm ^1^H/^13^C/^15^N E‐free magic‐angle spinning (MAS) probe. The sample temperature was set to 277 K. The MAS frequency was 12.5 kHz. 1D ^1^H‐^13^C cross‐polarization (CP) and 2D dipolar‐assisted rotational resonance experiments were acquired using a forward and back CP from ^1^H to ^13^C with a linear ramp from 49.0 to 61.2 kHz on ^1^H and constant ^13^C radiofrequency‐field amplitude at 80.5 kHz as well as high‐power heteronuclear decoupling at 83.3 kHz during acquisition. The CP contact time was 1 ms and the acquisition time was 10 ms. The ^13^C chemical shifts were referenced externally relative to adamantane (at 38.48 ppm relative to TMS). Spectra were processed with Bruker Topspin 4.0.

### Protein Expression Using a Bioreactor

A fed‐batch cultivation of *E. coli* for expression of (A_3_I)_3_‐A_14_ was performed as previously described for A_15_‐A_14_.^[^ ^56^ ^]^ Briefly, a preculture of BL21 (DE3) *E. coli* transformed for overexpressing (A_3_I)_3_‐A_14_ was grown in LB‐medium (50 µg mL^−1^ Kanamycin) at 37 °C. Once the OD_600_ reached ≈5, the preculture was used to inoculate (100‐fold dilution) fresh 250 mL cultivation medium (50 µg mL^−1^ Kanamycin, 0.01% antifoam 204) as defined by da Silva and coworkers.^[^ ^88^ ^]^ A Multifors 2 (Infors) equipped with a 0.5 L glass vessel was used to adjust the pH to 7, with 3 m H_3_PO_4_ and 25% NH_3_. The stirrer speed was adjusted automatically between 200 and 1200 rpm to obtain a dissolved relative oxygen level (pO2) of 30%. Initially the temperature was set to 28 °C, until the OD_600_ reached 50 (22 h after inoculation). Then the temperature was reduced to 20 °C before the culture was induced with IPTG to 150 µm. Feeding was initialized automatically 25 h after inoculation, using the cultivation medium with 40% glycerol, indicated by a sudden increase of pO_2_, and following an exponential feeding profile assuming a growth rate of μ = 0.1 h^−1^. Thus, the flow rate was varied between 2.8 and 20 mL h^−1^ until 125 mL of the feed stock solution were consumed. 20 h after induction, the culture was harvested by centrifugation at 4000 × *g*, the supernatant was discarded, and the cell pellet was resuspended in 20 mm Tris, pH 8 (20 mL/10 g wet cell pellet) and stored at −20 °C.

### Statistics

Data were analyzed on GraphPad prism, using one‐way ANOVA or multi variable analyses (correlation matrix with Pearson correlation coefficients) where appropriate. Statistical significance was indicated with asterisks: *p < 0.05; **p < 0.01; ***p < 0.001; ****p < 0.0001

## Conflict of Interest

The authors declare no conflict of interest.

## Supporting information

Supporting Information

Supplemental Video 1

## Data Availability

The data that support the findings of this study are available from the corresponding author upon reasonable request.
