# Supplementary material for: Engineered Spider Silk Proteins for Biomimetic Spinning of Fibers with Toughness Equal to Dragline Silks
Source: Adv Funct Mater. 2022 Mar 25;32(23):2200986. doi: 10.1002/adfm.202200986 (PMC9720699; doi:10.1002/adfm.202200986)
Supplement: Supplementary file 1 — Supporting Information [file ADFM-32-2200986-s002.pdf]

ADVANCED FUNCTIONAL MATERIALS

Supporting Information

for *Adv. Funct. Mater.,* DOI: 10.1002/adfm.202200986

Engineered Spider Silk Proteins for Biomimetic Spinning of Fibers with Toughness Equal to Dragline Silks

*Tina Arndt, Gabriele Greco, Benjamin Schmuck, Jessica Bunz, Olga Shilkova, Juanita Francis, Nicola M Pugno, Kristaps Jaudzems, Andreas Barth, Jan Johansson, and Anna Rising**

Supporting Information

Engineered Spider Silk Proteins for Biomimetic Spinning of Fibers with the Same Toughness as Dragline Silk

Tina Arndt[1], Gabriele Greco[2,3], Benjamin Schmuck[1,3], Jessica Bunz[1,4], Olga Shilkova[1], Juanita Francis[1], Nicola M Pugno[2,5], Kristaps Jaudzems[6], Andreas Barth[7], Jan Johansson[1], Anna Rising[1,3,*]

[1] Department of Biosciences and Nutrition, Karolinska Institutet, Neo, 14183 Huddinge, Sweden

[2] Laboratory for Bioinspired, Bionic, Nano, Meta, Materials & Mechanics, Department of Civil, Environmental and Mechanical Engineering, University of Trento, Via Mesiano 77, 38123 Trento, Italy.

[3] Department of Anatomy, Physiology and Biochemistry, Swedish University of Agricultural Sciences, 75007 Uppsala, Sweden

[4] Present address: Spiber Technologies AB, AlbaNova University Center, SE-10691 Stockholm, Sweden

[5] School of Engineering and Materials Sciences, Queen Mary University of London, Mile End Road, London E1 4NS, UK.

[6] Department of Physical Organic Chemistry, Latvian Institute of Organic Synthesis, Riga, LV-1006, Latvia.

[7] Department of Biochemistry and Biophysics, The Arrhenius Laboratories for Natural Sciences, Stockholm University, 10691 Stockholm, Sweden.

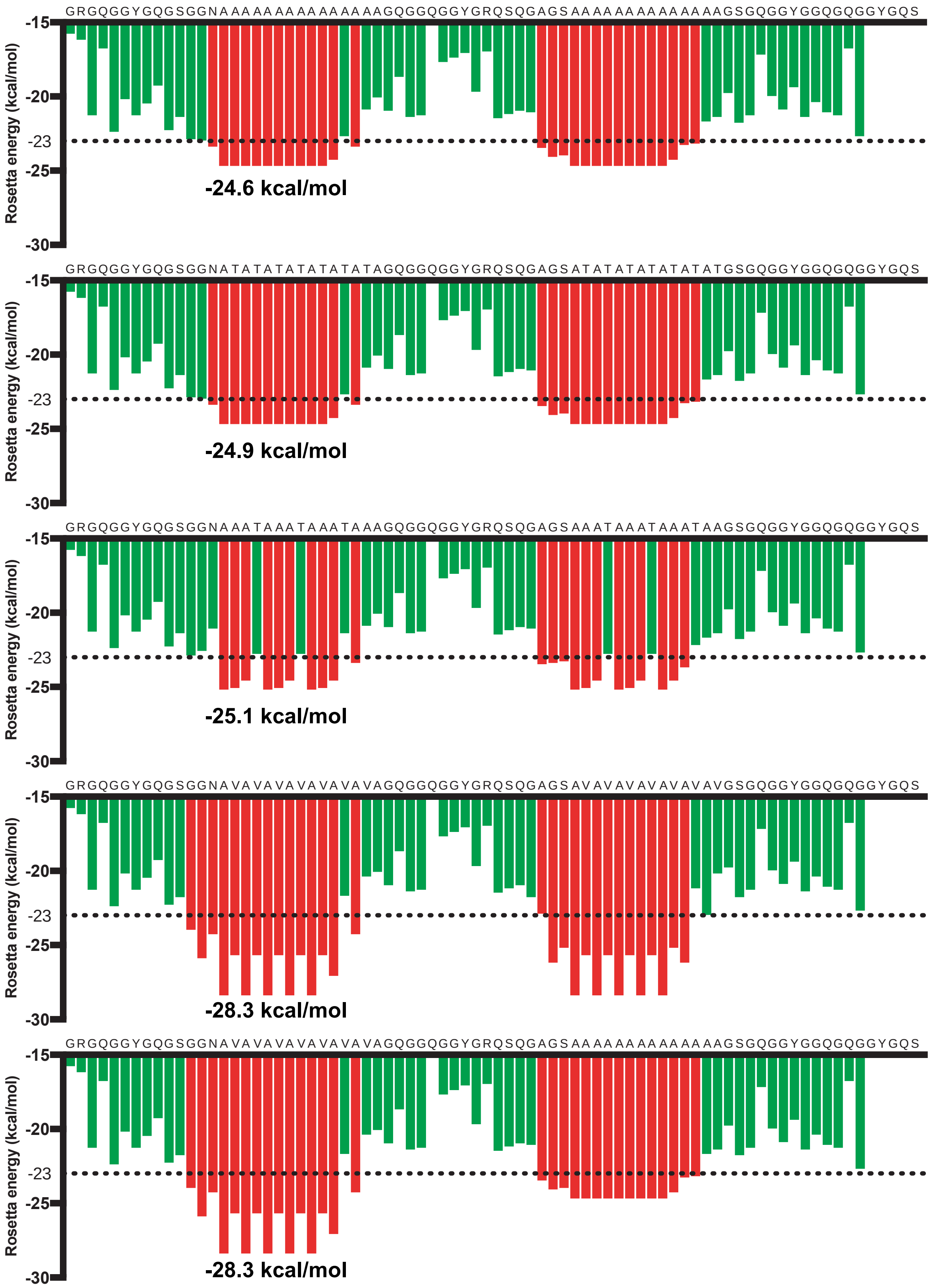
GRGQGGYGQGSGGNAAAAAAAAAAAAAAGQGGQGGYGRQSQGAGSAAAAAAAAAAAAAAGSGQGGYGGQGQGGYGQS
Rosetta energy (kcal/mol)
-15
-20
-23
-25
-30
-24.6 kcal/mol
GRGQGGYGQGSGGNATATATATATATATAGQGGQGGYGRQSQGAGSATATATATATATATGSGQGGYGGQGQGGYGQS
Rosetta energy (kcal/mol)
-15
-20
-23
-25
-30
-24.9 kcal/mol
GRGQGGYGQGSGGNAATAAATAAATAAAGQGGQGGYGRQSQGAGSAAATAAATAAATAAGSGQGGYGGQGQGGYGQS
Rosetta energy (kcal/mol)
-15
-20
-23
-25
-30
-25.1 kcal/mol
GRGQGGYGQGSGGNAVAVAVAVAVAVAVAGQGGQGGYGRQSQGAGSAVAVAVAVAVAVAVGSGQGGYGGQGQGGYGQS
Rosetta energy (kcal/mol)
-15
-20
-23
-25
-30
-28.3 kcal/mol
GRGQGGYGQGSGGNAVAVAVAVAVAVAVAGQGGQGGYGRQSQGAGSAAAAAAAAAAAAAAGSGQGGYGGQGQGGYGQS
Rosetta energy (kcal/mol)
-15
-20
-23
-25
-30
-28.3 kcal/mol

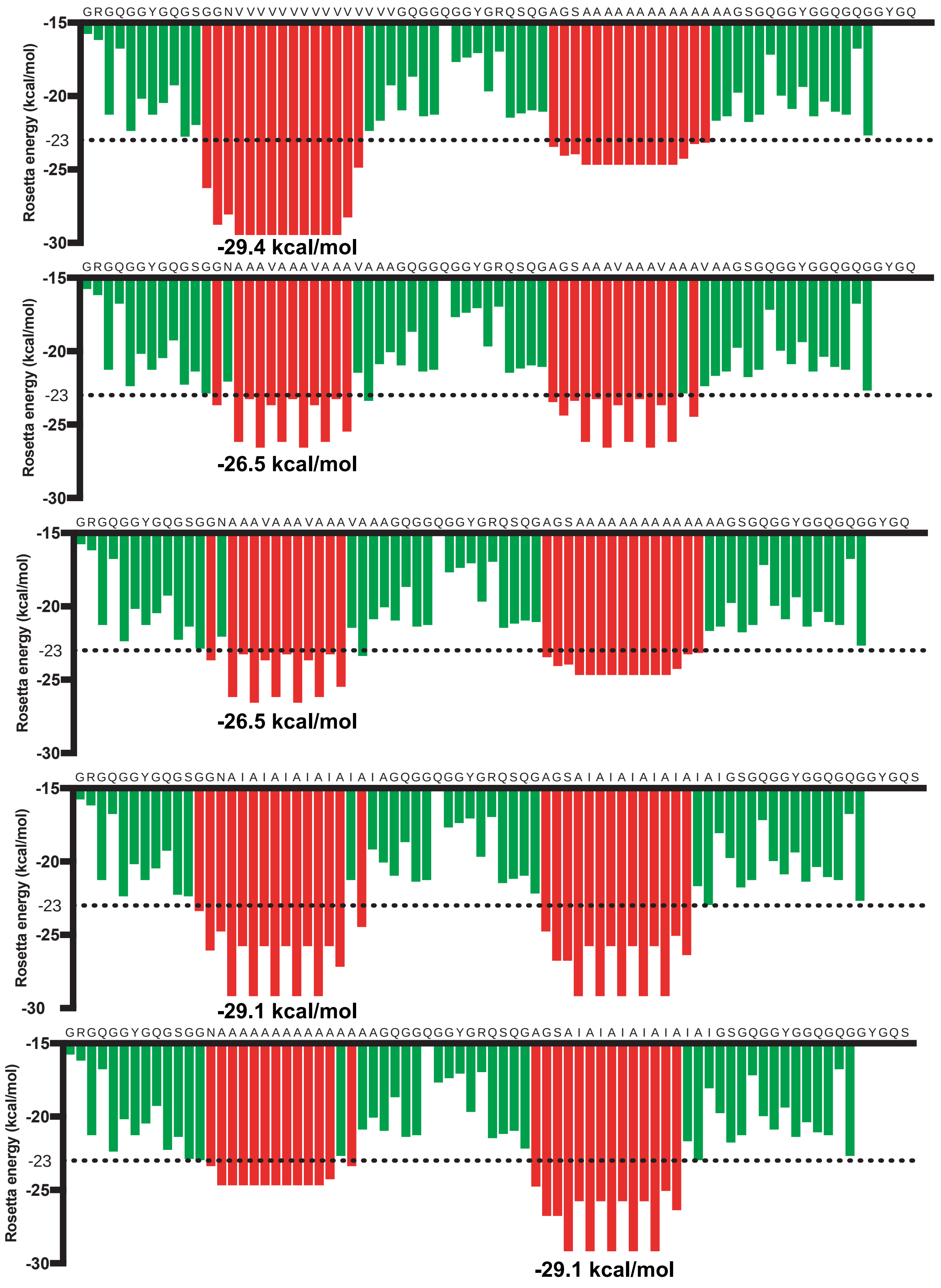
GRGQGGYGQGSGGNVVVVVVVVVVVVVVGQGGQGGYGRQSQGAGSAAAAAAAAAAAAAAGSGQGGYGGQGQGGYGQ
Rosetta energy (kcal/mol)
-15
-20
-23
-25
-30
-29.4 kcal/mol
GRGQGGYGQGSGGNAAAVAAAVAAAVAAAGQGGQGGYGRQSQGAGSAAAVAAAVAAAVAAGSGQGGYGGQGQGGYGQ
Rosetta energy (kcal/mol)
-15
-20
-23
-25
-30
-26.5 kcal/mol
GRGQGGYGQGSGGNAAAVAAAVAAAVAAAGQGGQGGYGRQSQGAGSAAAAAAAAAAAAAAGSGQGGYGGQGQGGYGQ
Rosetta energy (kcal/mol)
-15
-20
-23
-25
-30
-26.5 kcal/mol
GRGQGGYGQGSGGNAIAIAIAIAIAIAGQGGQGGYGRQSQGAGSAIAIAIAIAIAIGSGQGGYGGQGQGGYGQS
Rosetta energy (kcal/mol)
-15
-20
-23
-25
-30
-29.1 kcal/mol
GRGQGGYGQGSGGNAAAAAAAAAAAAAAAGQGGQGGYGRQSQGAGSAIAIAIAIAIAIGSGQGGYGGQGQGGYGQS
Rosetta energy (kcal/mol)
-15
-20
-23
-25
-30
-29.1 kcal/mol

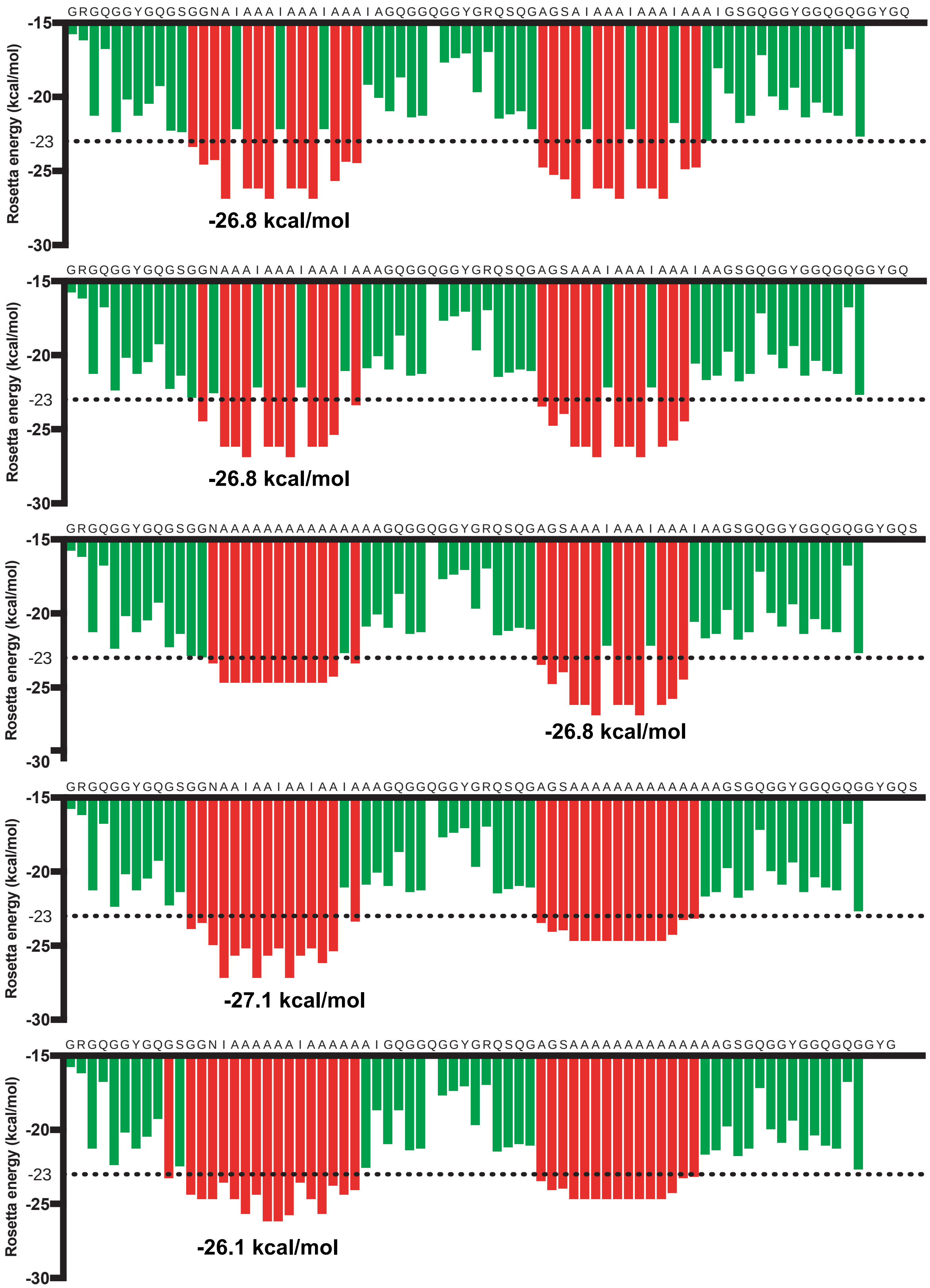

GRGQGGYGQGSGGNAIAAAIAAAIAAAIAGQGGQGGYGRQSQGAGSAIAAAIAAAIAAAIGSGQGGYGGQGQGGYGQ
Rosetta energy (kcal/mol)
-15
-20
-23
-25
-30
-26.8 kcal/mol
GRGQGGYGQGSGGNAAAIAAAIAAAIAAAGQGGQGGYGRQSQGAGSAAAIAAAIAAAIAAGSGQGGYGGQGQGGYGQ
Rosetta energy (kcal/mol)
-15
-20
-23
-25
-30
-26.8 kcal/mol
GRGQGGYGQGSGGNAAAAAAAAAAAAAAAGQGGQGGYGRQSQGAGSAAAIAAAIAAAIAAGSGQGGYGGQGQGGYGQS
Rosetta energy (kcal/mol)
-15
-20
-23
-25
-30
-26.8 kcal/mol
GRGQGGYGQGSGGNAAIAAIAAIAAIAAAGQGGQGGYGRQSQGAGSAAAAAAAAAAAAAAGSGQGGYGGQGQGGYGQS
Rosetta energy (kcal/mol)
-15
-20
-23
-25
-30
-27.1 kcal/mol
GRGQGGYGQGSGGNIAAAAAAIAAAAAAIGQGGQGGYGRQSQGAGSAAAAAAAAAAAAAAGSGQGGYGGQGQGGYG
Rosetta energy (kcal/mol)
-15
-20
-23
-25
-30
-26.1 kcal/mol

Figure S 1: Rosetta energy profiles of all engineered mini-spidroins. Bars show Rosetta energies for moving hexapeptides (indicated at the first residue of each hexapeptide), red bars indicate Rosetta energies equal or below -23 kcal/mol (dashed line). Green bars indicate Rosetta energies above the threshold and are unlikely to form steric zippers (https://services.mbi.ucla.edu/zipperdb/). Lowest Rosetta energies are indicated.

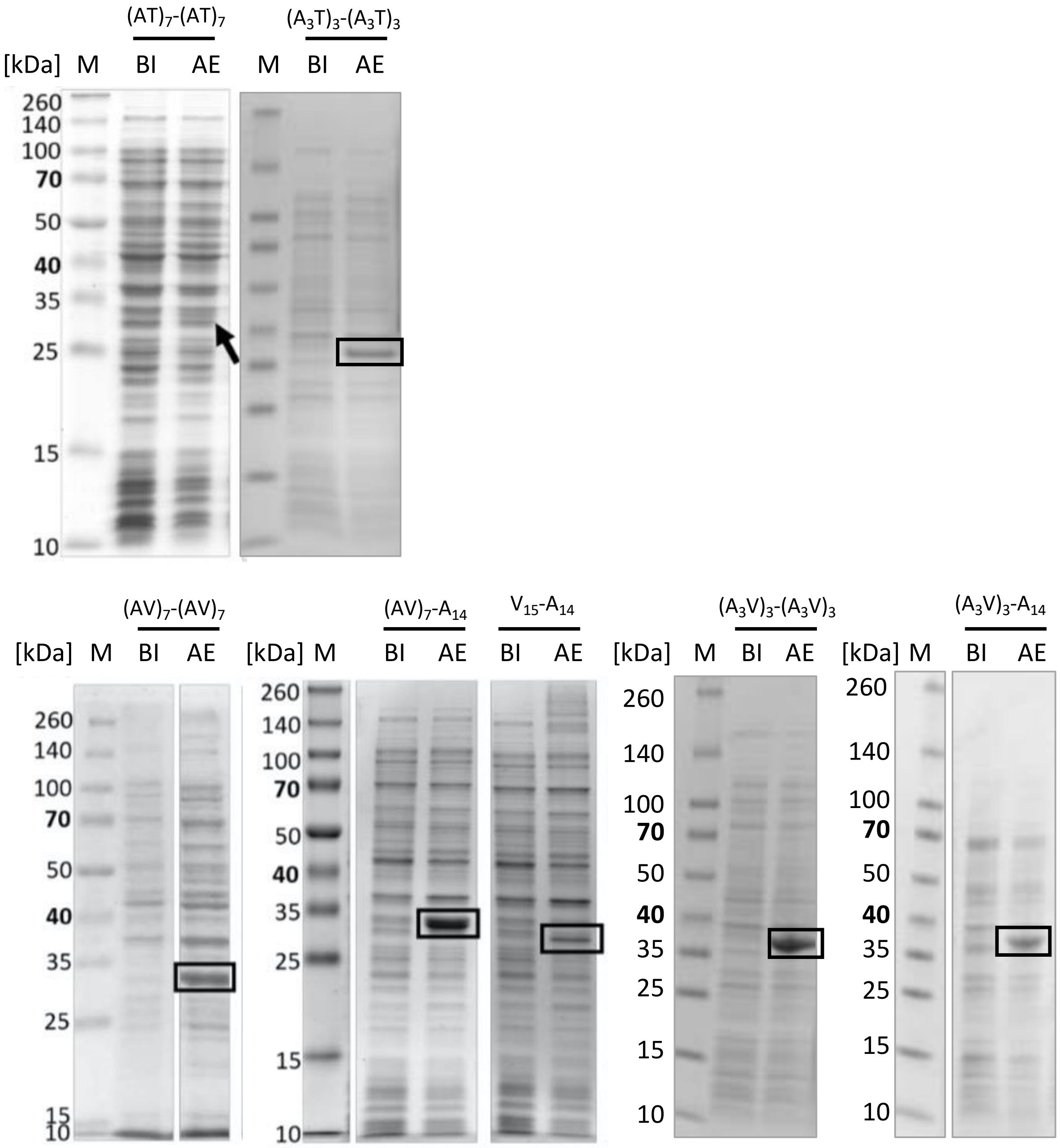

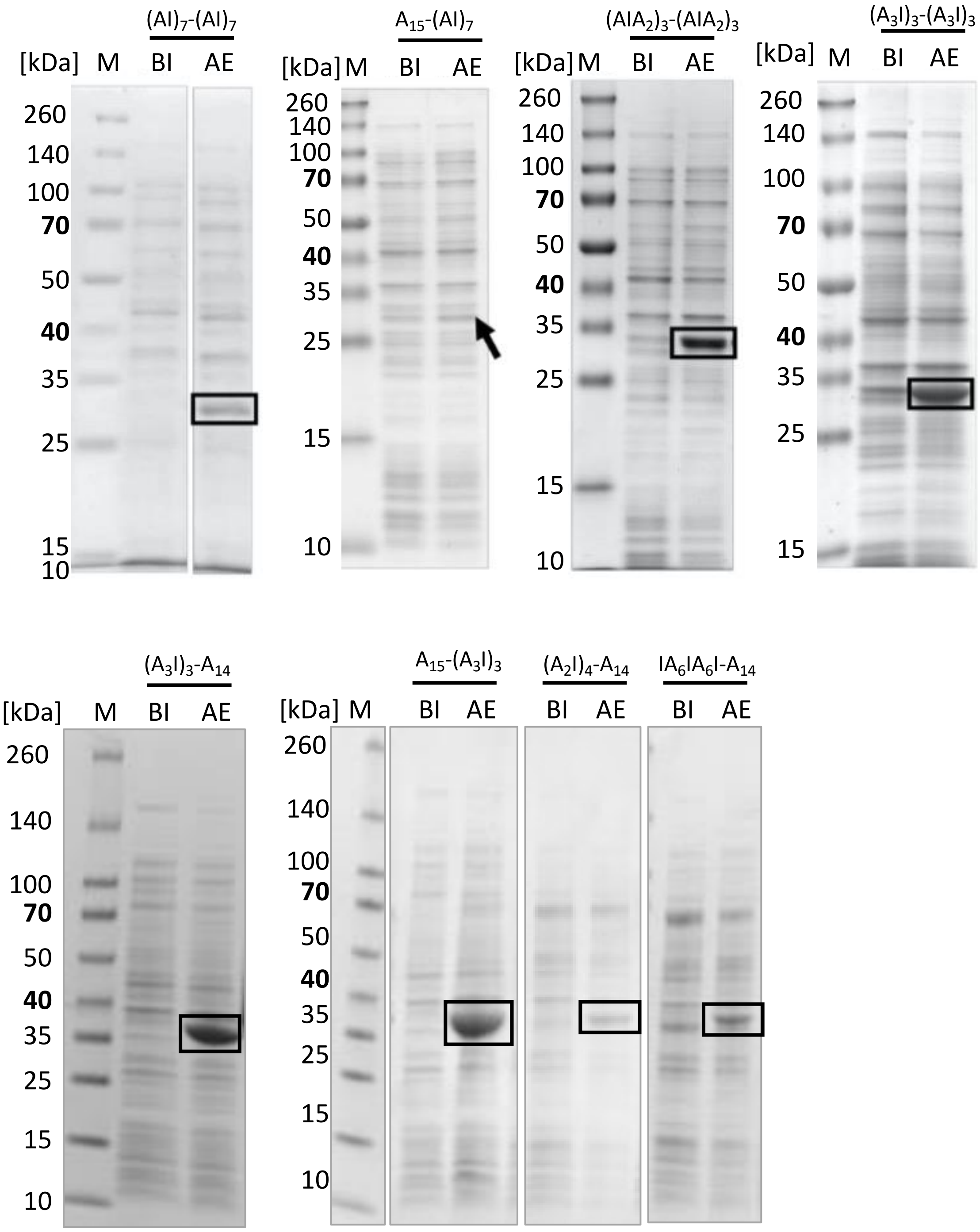

Figure S 2: Expression levels of constructs. Constructs with Thr, Val, and Ile substitutions. M- marker, BI- before induction, AE- after expression, box indicates target protein.

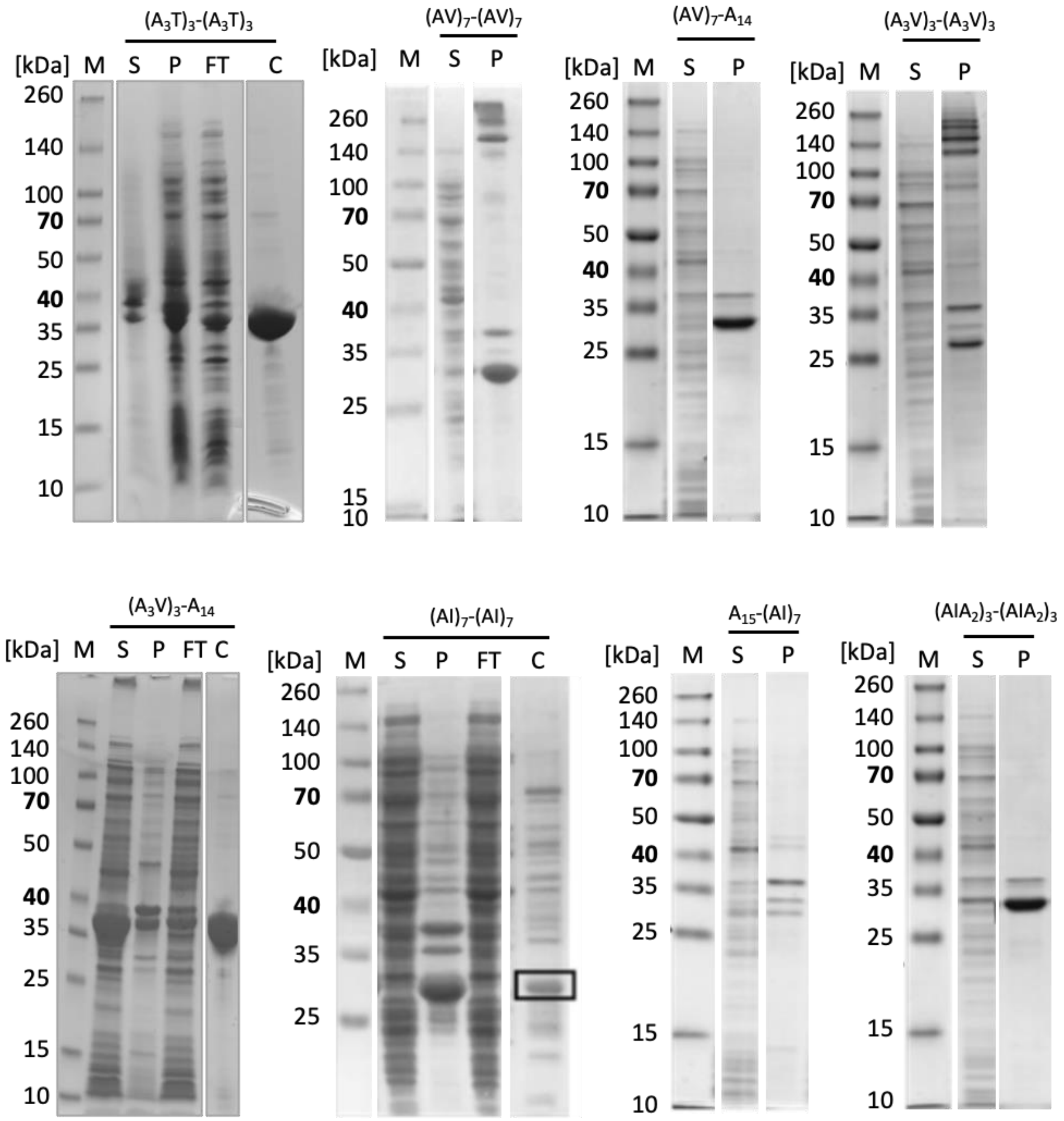
(A3T)3-(A3T)3
[kDa] M S P FT C
260 140 100 70 50 40 35 25 15 10
(AV)7-(AV)7
[kDa] M S P
260 140 100 70 50 40 35 25 15 10
(AV)7-A14
[kDa] M S P
260 140 100 70 50 40 35 25 15 10
(A3V)3-(A3V)3
[kDa] M S P
260 140 100 70 50 40 35 25 15 10
(A3V)3-A14
[kDa] M S P FT C
260 140 100 70 50 40 35 25 15 10
(AI)7-(AI)7
[kDa] M S P FT C
260 140 100 70 50 40 35 25
A15-(AI)7
[kDa] M S P
260 140 100 70 50 40 35 25 15 10
(AIA2)3-(AIA2)3
[kDa] M S P
260 140 100 70 50 40 35 25 15 10

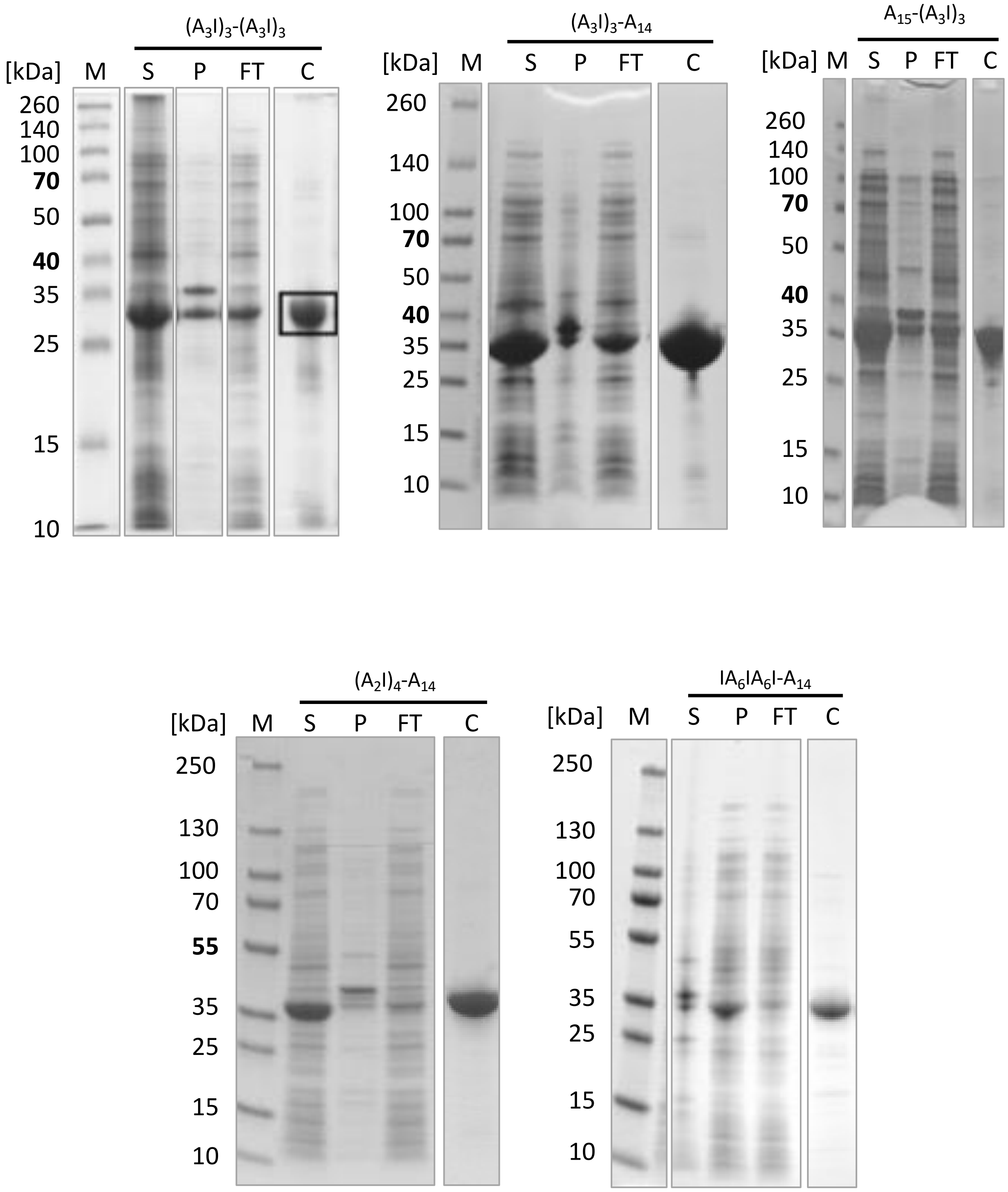

Figure S 3: Solubility and purification of constructs evaluated by SDS-PAGE. M- marker, S- soluble fraction, P-pellet (resuspended), FT- flow-through the IMAC, C-target construct.

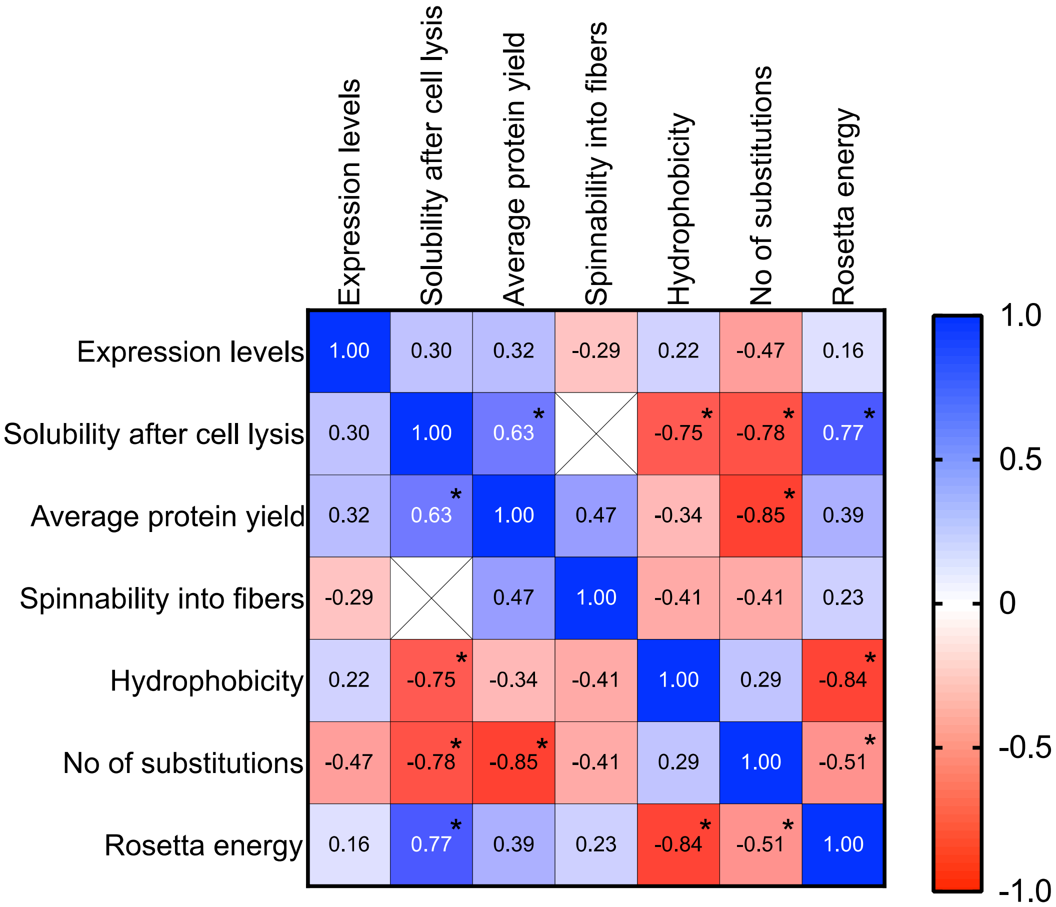

Figure S 4: Pearson correlation matrix (r values) of expression levels, solubility, yield, spinnability, hydrophobicity (only the repeat region), number of substitutions and Rosetta energies. Solubility, expression levels and spinnability were rated from very high (3) to none (0), (according to Table 2). * indicates statistical significance ($p < 0.05$).

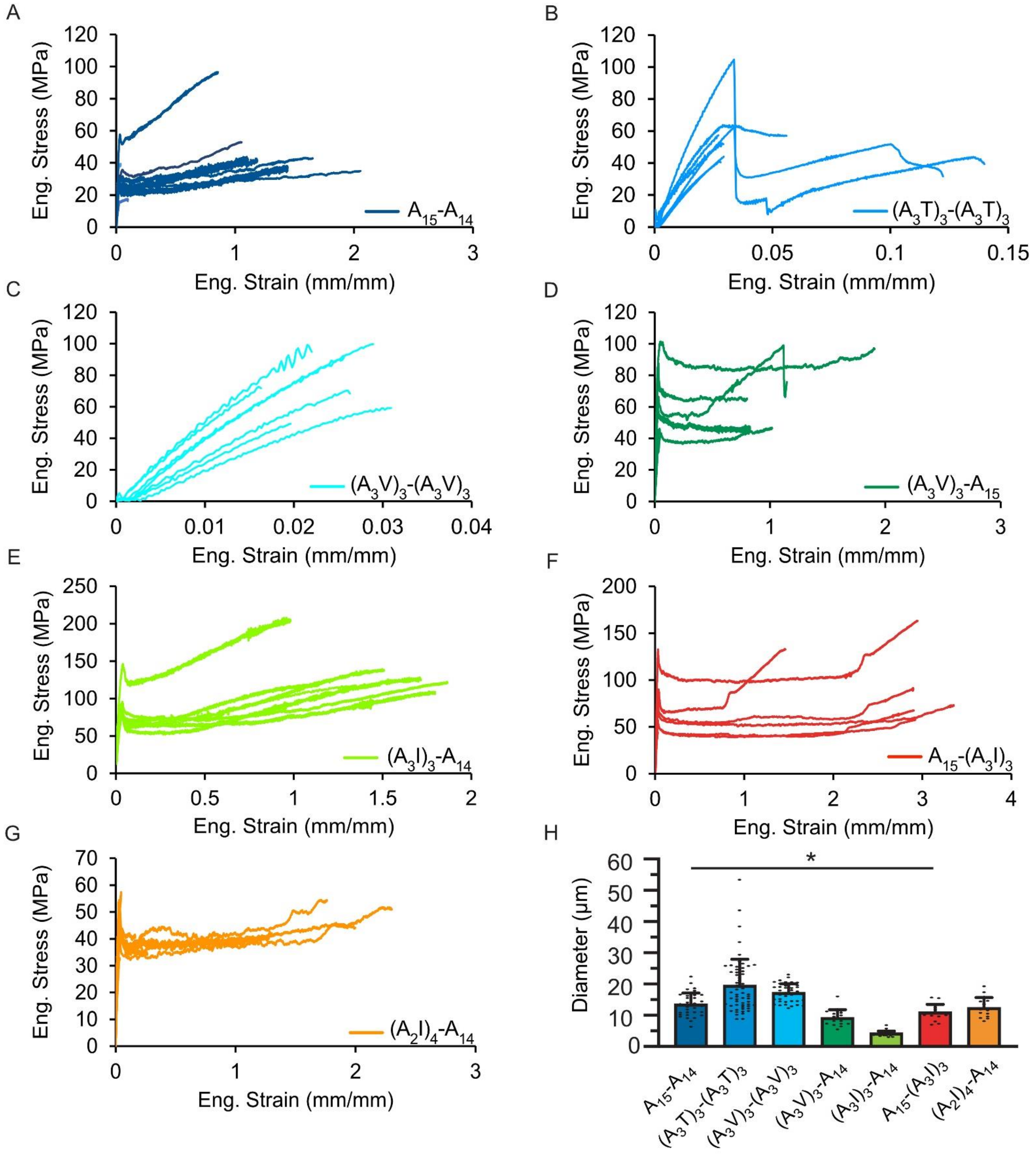

Figure S 5: Mechanical properties of spinnable constructs continued. A-G) Representative stress-strain curves of all engineered proteins that were spun into fibers. Each panel shows 8 stress strain curves. H) diameter of the fibers.

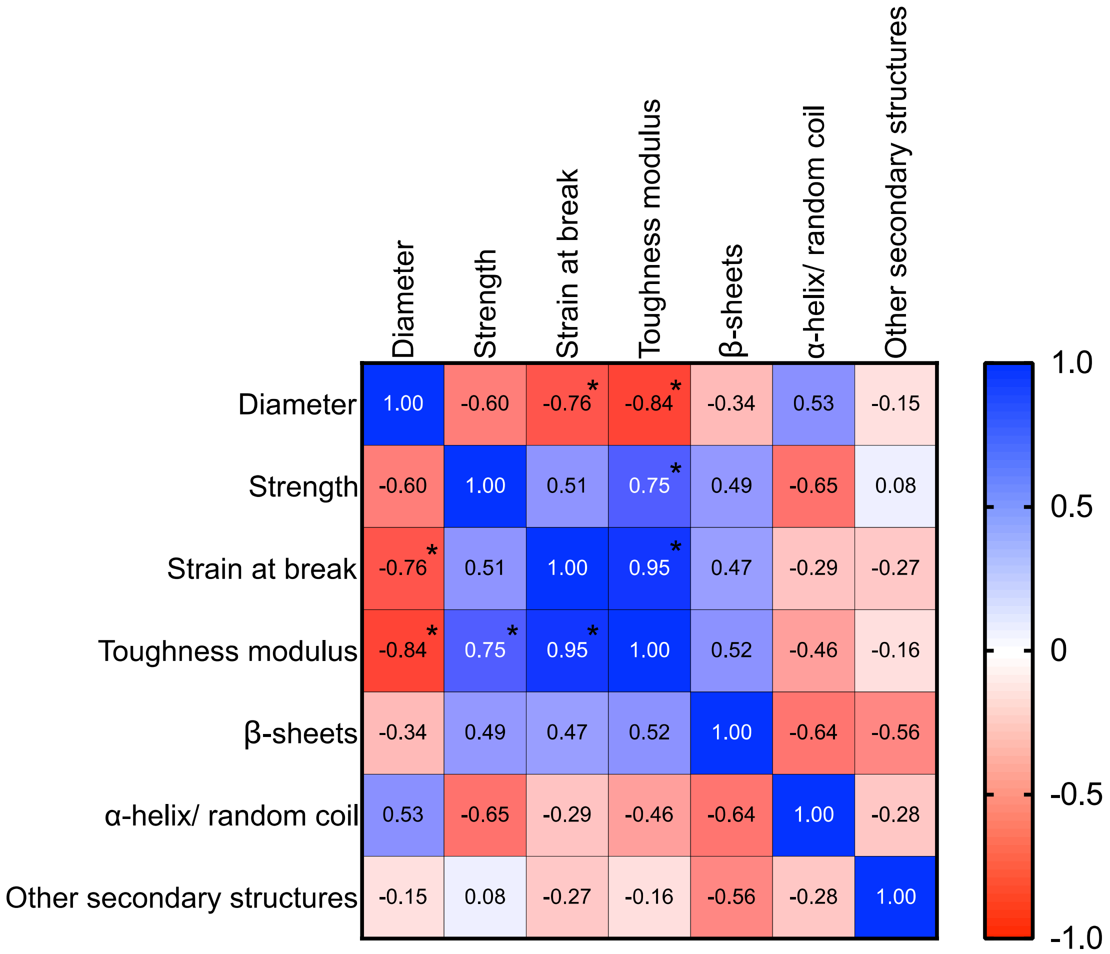

Figure S 6: Pearson correlation matrix (r values) of mechanical properties and secondary structure content. * indicates statistical significance ($p < 0.05$).

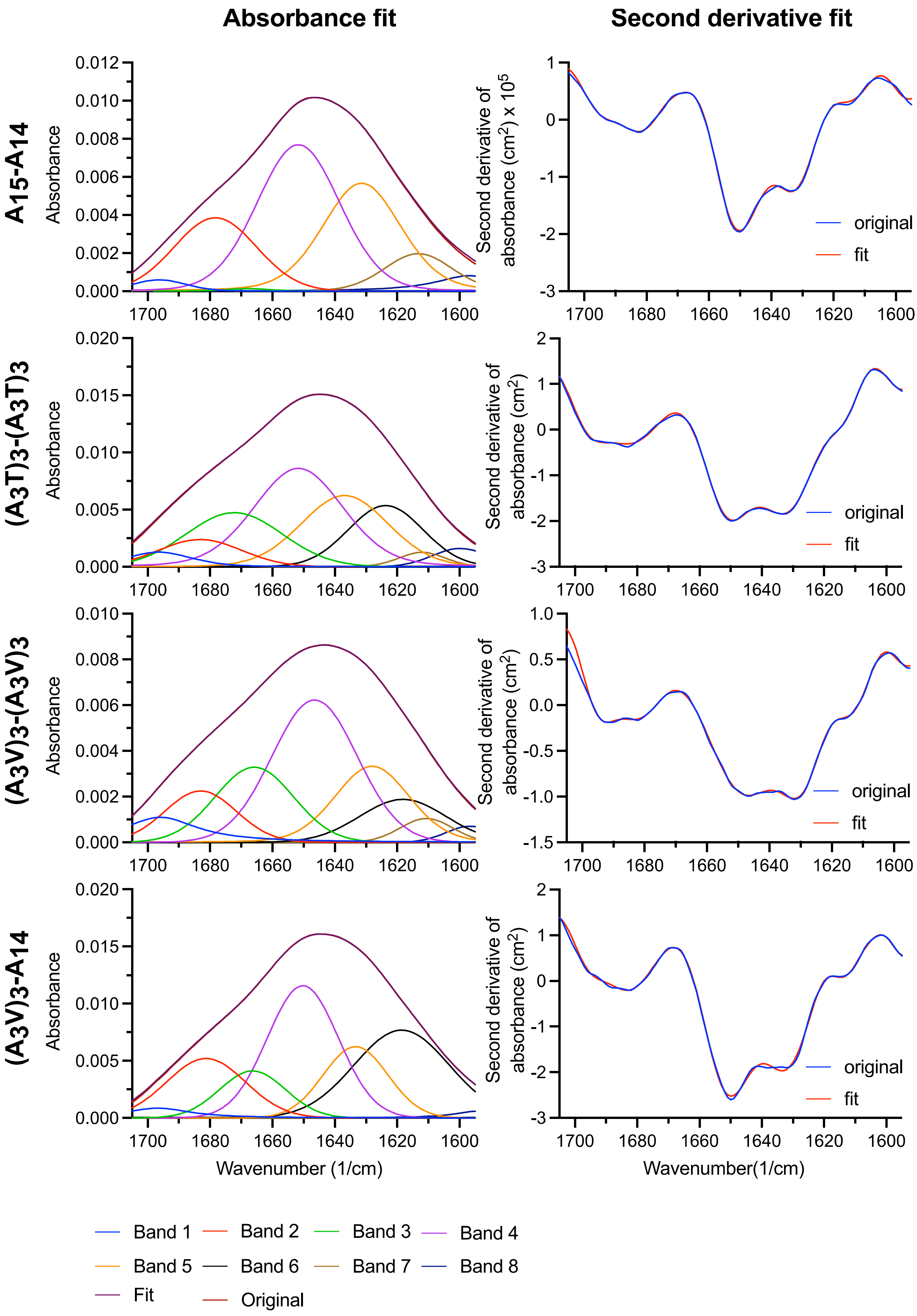

Absorbance fit
Second derivative fit
A15-A14
(A3T)3-(A3T)3
(A3V)3-(A3V)3
(A3V)3-A14
Absorbance
Second derivative of absorbance (cm2) x 105
Second derivative of absorbance (cm2)
original
fit
Wavenumber (1/cm)
Wavenumber(1/cm)
Band 1
Band 2
Band 3
Band 4
Band 5
Band 6
Band 7
Band 8
Fit
Original

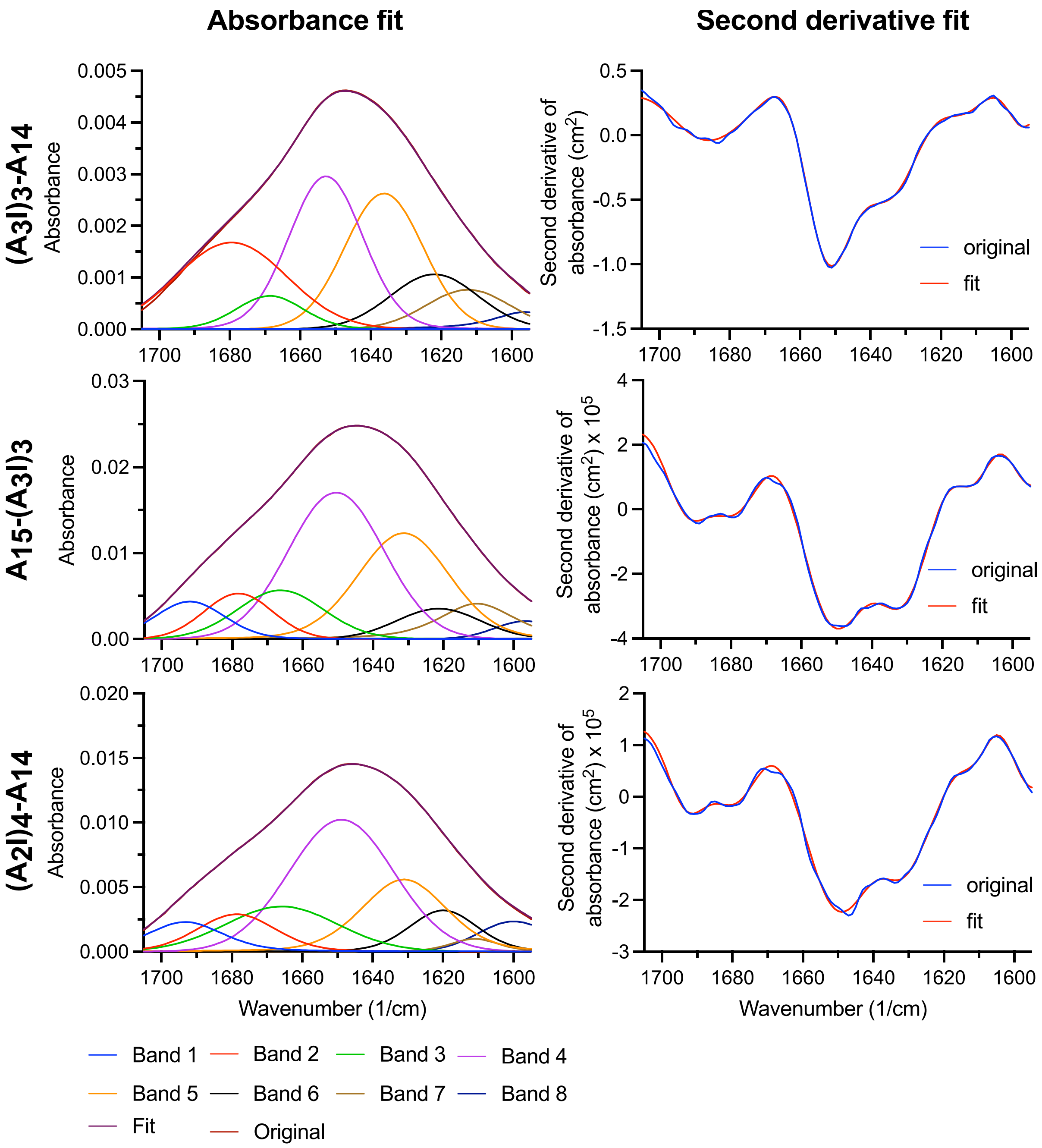

Figure S 7: Fits of absorbance spectra and second derivative of fibers spun from engineered mini-spidroins. Band 1, 5, 6, 7: b-sheets; Band 2, 3: others; Band 4: a-helix/ random-coil; Band 8: side chains.

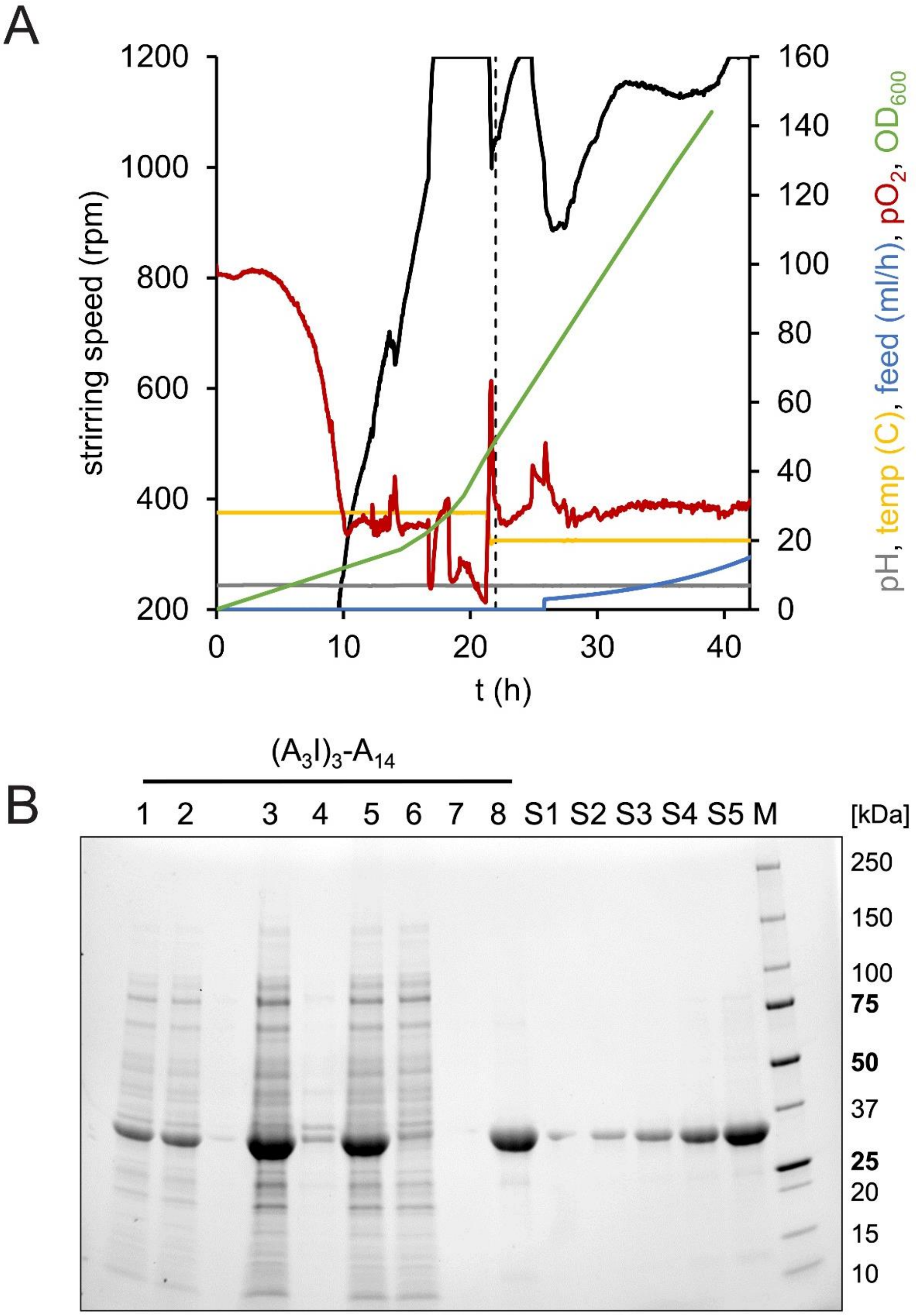

Figure S 8: Expression and purification of $(A_3I)_3$-A_{14} produced in a bioreactor-based E. coli fed batch culture. B) Cultivation parameters for the expression of $(A_3I)_3$-A_{14}. The stirring speed (black), pO2 (red), pH (grey), temperature (orange), feeding rate (blue), and the optical cell density (green) are shown. The induction point (22 h after inoculation) is indicated by a black dashed line. B) SDS PAGE of lane 1: total cell content 18.5 h after induction (20-fold dilution); lane 2: total cell content 21.5 h after induction (20-fold dilution); lane 3: total cell content after cell lysis; lane 4: pellet after centrifugation; lane 5: cell lysate; lane 6: flow-through; lane 7: wash using 5 mM imidazole; lane 8: eluate using 200 mM imidazole (5-fold dilution). S1-S5 reference samples of A_{15}-A_{14}. S1: 0.112 mg/mL; S2: 0.225 mg/mL; S3: 0.45 mg/mL; S4: 0.9 mg/mL; S5: 1.8 mg/mL.

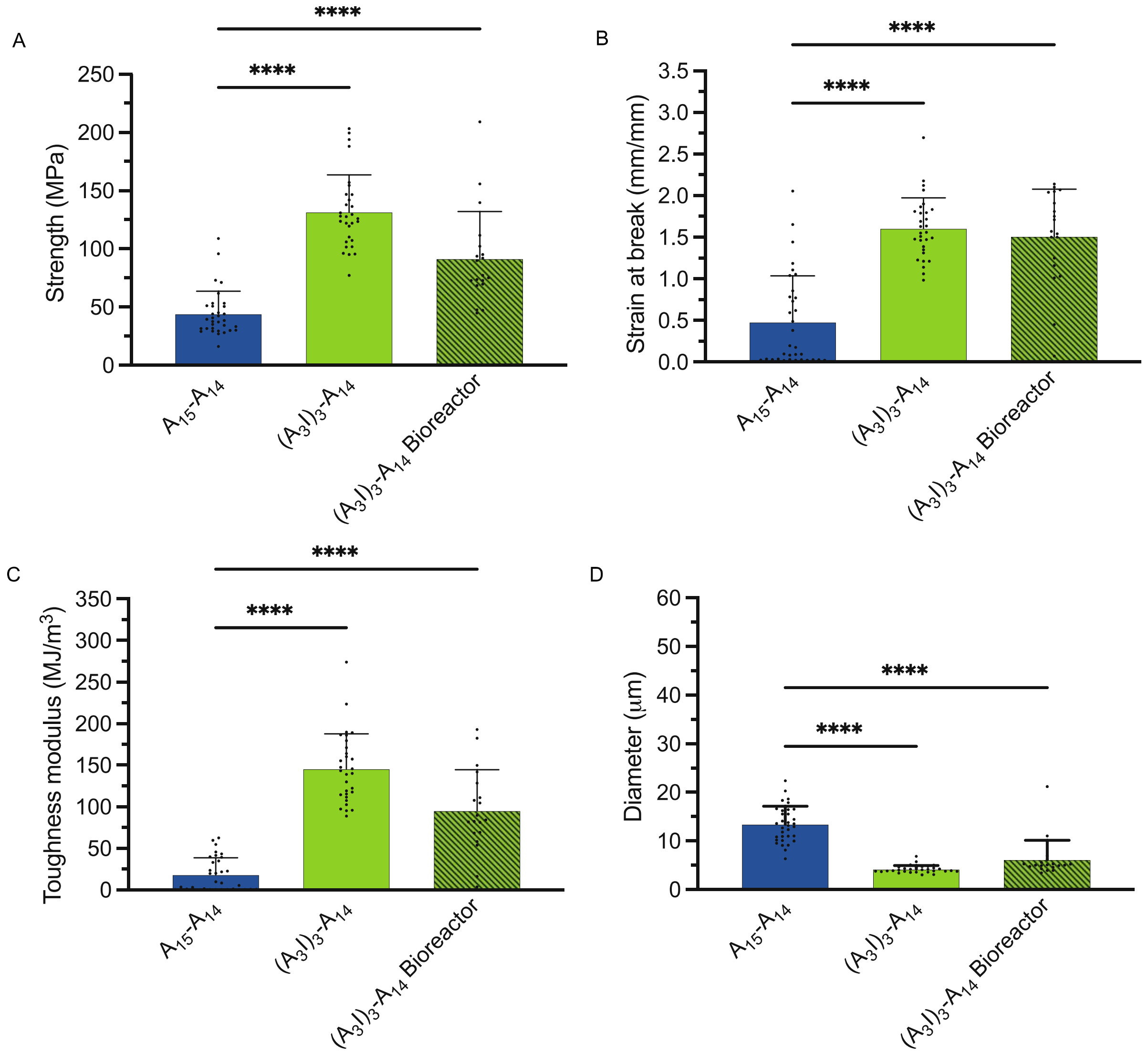

Figure S 9: Mechanical properties of fibers produced from mini-spidroins expressed using a shake-flask or a bioreactor. A) Strength, B) strain at break, C) toughness modulus and D) diameter. Whiskers show standard deviation. **** $p < 0.0001$

Table S 1: Amino acid sequences of A_{15}-A_{14} and engineered constructs thereof:

**Name**	**Amino acid sequence**
A_{15}-A_{14}	MGHHHHHHMSHTTPWTNPGLAENFMNSFMQGLSSMPGFTASQLDDMST<br>IAQSMVQSIQSLAAQGRTSPNKLQALNMAFASSMAEIAASEEGGGSLSTKTSS<br>IASAMSNAFLQTTGVVNQPFINEITQLVSMFAQAGMNDVSAGNSGRGQGG<br>YGQGSGGNAAAAAAAAAAAAAAAGQGGQGGYGRQSQGAGSAAAAAAAA<br>AAAAAAGSGQGGYGGQGQGGYGQSGNSVTSGGYGYGTSAAAGAGVAAGS<br>YAGAVNRLSSAEAASRVSSNIAAIASGGASALPSVISNIYSGVVASGVSSNEALI<br>QALLELLSALVHVLSSASIGNVSSVGVDSTLNVVQDSVGQYVG*
$(AT)_7$-$(AT)_7$	MGHHHHHHMSHTTPWTNPGLAENFMNSFMQGLSSMPGFTASQLDDMST<br>IAQSMVQSIQSLAAQGRTSPNKLQALNMAFASSMAEIAASEEGGGSLSTKTSS<br>IASAMSNAFLQTTGVVNQPFINEITQLVSMFAQAGMNDVSAGNSGRGQGG<br>YGQGSGGNATATATATATATATAGQGGQGGYGRQSQGAGSATATATATAT<br>ATATGSGQGGYGGQGQGGYGQSGNSVTSGGYGYGTSAAAGAGVAAGSYA<br>GAVNRLSSAEAASRVSSNIAAIASGGASALPSVISNIYSGVVASGVSSNEALIQA<br>LLELLSALVHVLSSASIGNVSSVGVDSTLNVVQDSVGQYVG*
$(A_3T)_3$-$(A_3T)_3$	MGHHHHHHMSHTTPWTNPGLAENFMNSFMQGLSSMPGFTASQLDDMST<br>IAQSMVQSIQSLAAQGRTSPNKLQALNMAFASSMAEIAASEEGGGSLSTKTSS<br>IASAMSNAFLQTTGVVNQPFINEITQLVSMFAQAGMNDVSAGNSGRGQGG<br>YGQGSGGNAAATAAATAAATAAAGQGGQGGYGRQSQGAGSAAATAAATA<br>AATAAGSGQGGYGGQGQGGYGQSGNSVTSGGYGYGTSAAAGAGVAAGSY<br>AGAVNRLSSAEAASRVSSNIAAIASGGASALPSVISNIYSGVVASGVSSNEALIQ<br>ALLELLSALVHVLSSASIGNVSSVGVDSTLNVVQDSVGQYVG*
$(AV)_7$-$(AV)_7$	MGHHHHHHMSHTTPWTNPGLAENFMNSFMQGLSSMPGFTASQLDDMST<br>IAQSMVQSIQSLAAQGRTSPNKLQALNMAFASSMAEIAASEEGGGSLSTKTSS<br>IASAMSNAFLQTTGVVNQPFINEITQLVSMFAQAGMNDVSAGNSGRGQGG<br>YGQGSGGNAVAVAVAVAVAVAVAGQGGQGGYGRQSQGAGSAVAVAVAV<br>AVAVAVGSGQGGYGGQGQGGYGQSGNSVTSGGYGYGTSAAAGAGVAAGS<br>YAGAVNRLSSAEAASRVSSNIAAIASGGASALPSVISNIYSGVVASGVSSNEALI<br>QALLELLSALVHVLSSASIGNVSSVGVDSTLNVVQDSVGQYVG*
$(AV)_7$-A_{14}	MGHHHHHHMSHTTPWTNPGLAENFMNSFMQGLSSMPGFTASQLDDMST<br>IAQSMVQSIQSLAAQGRTSPNKLQALNMAFASSMAEIAASEEGGGSLSTKTSS<br>IASAMSNAFLQTTGVVNQPFINEITQLVSMFAQAGMNDVSAGNSGRGQGG<br>YGQGSGGNAVAVAVAVAVAVAVAGQGGQGGYGRQSQGAGSAAAAAAAA<br>AAAAAAGSGQGGYGGQGQGGYGQSGNSVTSGGYGYGTSAAAGAGVAAGS<br>YAGAVNRLSSAEAASRVSSNIAAIASGGASALPSVISNIYSGVVASGVSSNEALI<br>QALLELLSALVHVLSSASIGNVSSVGVDSTLNVVQDSVGQYVG*
V_{15}-A_{14}	MGHHHHHHMSHTTPWTNPGLAENFMNSFMQGLSSMPGFTASQLDDMST<br>IAQSMVQSIQSLAAQGRTSPNKLQALNMAFASSMAEIAASEEGGGSLSTKTSS<br>IASAMSNAFLQTTGVVNQPFINEITQLVSMFAQAGMNDVSAGNSGRGQGG<br>YGQGSGGNVVVVVVVVVVVVVVVGQGGQGGYGRQSQGAGSAAAAAAAA<br>AAAAAAGSGQGGYGGQGQGGYGQSGNSVTSGGYGYGTSAAAGAGVAAGS<br>YAGAVNRLSSAEAASRVSSNIAAIASGGASALPSVISNIYSGVVASGVSSNEALI<br>QALLELLSALVHVLSSASIGNVSSVGVDSTLNVVQDSVGQYVG*
$(A_3V)_3$-$(A_3V)_3$	MGHHHHHHMSHTTPWTNPGLAENFMNSFMQGLSSMPGFTASQLDDMST<br>IAQSMVQSIQSLAAQGRTSPNKLQALNMAFASSMAEIAASEEGGGSLSTKTSS

	IASAMSNAFLQTTGVVNQPFINEITQLVSMFAQAGMNDVSAGNSGRGQGG YGQGSGGNAAAVAAAVAAAVAAAGQGGQGGYGRQSQGAGSAAAVAAAV AAAVAAGSGQGGYGGQGQGGYGQSGNSVTSGGYGYGTSAAAGAGVAAGS YAGAVNRLSSAEAASRVSSNIAAIASGGASALPSVISNIYSGVVASGVSSNEALI QALLELLSALVHVLSSASIGNVSSVGVDSTLNVVQDSVGQYVG*
$(A_3V)_3$-A_{14}	MGHHHHHHMSHTTPWTNPGLAENFMNSFMQGLSSMPGFTASQLDDMST IAQSMVQSIQSLAAQGRTSPNKLQALNMAFASSMAEIAASEEGGGSLSTKTSS IASAMSNAFLQTTGVVNQPFINEITQLVSMFAQAGMNDVSAGNSGRGQGG YGQGSGGNAAAVAAAVAAAVAAAGQGGQGGYGRQSQGAGSAAAAAAAA AAAAAAGSGQGGYGGQGQGGYGQSVTSGGYGYGTSAAAGAGVAAGSYAG AVNRLSSAEAASRVSSNIAAIASGGASALPSVISNIYSGVVASGVSSNEALIQALL ELLSALVHVLSSASIGNVSSVGVDSTLNVVQDSVGQYVG*
$(AI)_7$-$(AI)_7$	MGHHHHHHMSHTTPWTNPGLAENFMNSFMQGLSSMPGFTASQLDDMST IAQSMVQSIQSLAAQGRTSPNKLQALNMAFASSMAEIAASEEGGGSLSTKTSS IASAMSNAFLQTTGVVNQPFINEITQLVSMFAQAGMNDVSAGNSGRGQGG YGQGSGGNAIAIAIAIAIAIAIAGQGGQGGYGRQSQGAGSAIAIAIAIAIAIAIGS GQGGYGGQGQGGYGQSGNSVTSGGYGYGTSAAAGAGVAAGSYAGAVNRL SSAEAASRVSSNIAAIASGGASALPSVISNIYSGVVASGVSSNEALIQALLELLSAL VHVLSSASIGNVSSVGVDSTLNVVQDSVGQYVG*
A_{15}-$(AI)_7$	MGHHHHHHMSHTTPWTNPGLAENFMNSFMQGLSSMPGFTASQLDDMST IAQSMVQSIQSLAAQGRTSPNKLQALNMAFASSMAEIAASEEGGGSLSTKTSS IASAMSNAFLQTTGVVNQPFINEITQLVSMFAQAGMNDVSAGNSGRGQGG YGQGSGGNAAAAAAAAAAAAAAAGQGGQGGYGRQSQGAGSAIAIAIAIAIA IAIGSGQGGYGGQGQGGYGQSGNSVTSGGYGYGTSAAAGAGVAAGSYAGA VNRLSSAEAASRVSSNIAAIASGGASALPSVISNIYSGVVASGVSSNEALIQALLE LLSALVHVLSSASIGNVSSVGVDSTLNVVQDSVGQYVG*
$(AIA_2)_3$-$(AIA_2)_3$	MGHHHHHHMSHTTPWTNPGLAENFMNSFMQGLSSMPGFTASQLDDMST IAQSMVQSIQSLAAQGRTSPNKLQALNMAFASSMAEIAASEEGGGSLSTKTSS IASAMSNAFLQTTGVVNQPFINEITQLVSMFAQAGMNDVSAGNSGRGQGG YGQGSGGNAIAAAIAAAIAAAIAGQGGQGGYGRQSQGAGSAIAAAIAAAIAA AIGSGQGGYGGQGQGGYGQSGNSVTSGGYGYGTSAAAGAGVAAGSYAGA VNRLSSAEAASRVSSNIAAIASGGASALPSVISNIYSGVVASGVSSNEALIQALLE LLSALVHVLSSASIGNVSSVGVDSTLNVVQDSVGQYVG*
$(A_3I)_3$-$(A_3I)_3$	MGHHHHHHMSHTTPWTNPGLAENFMNSFMQGLSSMPGFTASQLDDMST IAQSMVQSIQSLAAQGRTSPNKLQALNMAFASSMAEIAASEEGGGSLSTKTSS IASAMSNAFLQTTGVVNQPFINEITQLVSMFAQAGMNDVSAGNSGRGQGG YGQGSGGNAAAIAAAIAAAIAAAGQGGQGGYGRQSQGAGSAAAIAAAIAAA IAAGSGQGGYGGQGQGGYGQSGNSVTSGGYGYGTSAAAGAGVAAGSYAG AVNRLSSAEAASRVSSNIAAIASGGASALPSVISNIYSGVVASGVSSNEALIQALL ELLSALVHVLSSASIGNVSSVGVDSTLNVVQDSVGQYVG*
$(A_3I)_3$-A_{14}	MGHHHHHHMSHTTPWTNPGLAENFMNSFMQGLSSMPGFTASQLDDMST IAQSMVQSIQSLAAQGRTSPNKLQALNMAFASSMAEIAASEEGGGSLSTKTSS IASAMSNAFLQTTGVVNQPFINEITQLVSMFAQAGMNDVSAGNSGRGQGG YGQGSGGNAAAIAAAIAAAIAAAGQGGQGGYGRQSQGAGSAAAAAAAAAA AAAAGSGQGGYGGQGQGGYGQSGNSVTSGGYGYGTSAAAGAGVAAGSYA GAVNRLSSAEAASRVSSNIAAIASGGASALPSVISNIYSGVVASGVSSNEALIQA LLELLSALVHVLSSASIGNVSSVGVDSTLNVVQDSVGQYVG*

A_{15}-$(A_3I)_3$	MGHHHHHHMSHTTPWTNPGLAENFMNSFMQGLSSMPGFTASQLDDMST IAQSMVQSIQSLAAQGRTSPNKLQALNMAFASSMAEIAASEEGGGSLSTKTSS IASAMSNAFLQTTGVVNQPFINEITQLVSMFAQAGMNDVSAGNSGRGQGG YGQGSGGNAAAAAAAAAAAAAAAGQGGQGGYGRQSQGAGSAAAIAAAIA AAIAAGSGQGGYGGQGQGGYGQSGNSVTSGGYGYGTSAAAGAGVAAGSYA GAVNRLSSAEAASRVSSNIAAIASGGASALPSVISNIYSGVVASGVSSNEALIQA LLELLSALVHVLSSASIGNVSSVGVDSTLNVVQDSVGQYVG*
$(A_2I)_4$-A_{14}	MGHHHHHHMSHTTPWTNPGLAENFMNSFMQGLSSMPGFTASQLDDMST IAQSMVQSIQSLAAQGRTSPNKLQALNMAFASSMAEIAASEEGGGSLSTKTSS IASAMSNAFLQTTGVVNQPFINEITQLVSMFAQAGMNDVSAGNSGRGQGG YGQGSGGNAAIAAIAAIAAIAAAGQGGQGGYGRQSQGAGSAAAAAAAAAA AAAAGSGQGGYGGQGQGGYGQSGNSVTSGGYGYGTSAAAGAGVAAGSYA GAVNRLSSAEAASRVSSNIAAIASGGASALPSVISNIYSGVVASGVSSNEALIQA LLELLSALVHVLSSASIGNVSSVGVDSTLNVVQDSVGQYVG*
IA_6IA_6I-A_{14}	MGHHHHHHMSHTTPWTNPGLAENFMNSFMQGLSSMPGFTASQLDDMST IAQSMVQSIQSLAAQGRTSPNKLQALNMAFASSMAEIAASEEGGGSLSTKTSS IASAMSNAFLQTTGVVNQPFINEITQLVSMFAQAGMNDVSAGNSGRGQGG YGQGSGGNIAAAAAAIAAAAAAIGQGGQGGYGRQSQGAGSAAAAAAAAAA AAAAGSGQGGYGGQGQGGYGQSGNSVTSGGYGYGTSAAAGAGVAAGSYA GAVNRLSSAEAASRVSSNIAAIASGGASALPSVISNIYSGVVASGVSSNEALIQA LLELLSALVHVLSSASIGNVSSVGVDSTLNVVQDSVGQYVG*

Table S 2: Hexapeptides with lowest Rosetta energies and hydropathy of the engineered mini-spidroins.

Construct	Example of hexapeptide	Rosetta energy (kcal/mol)	Hydropathy
A_{15}-A_{14}	AAAAAA	-24.6	-0.168
$(AT)_7$-$(AT)_7$	ATATAT	-24.9	-0.617
$(A_3T)_3$-$(A_3T)_3$	AAATAA	-25.1	-0.36
$(AV)_7$-$(AV)_7$	AVAVAV	-28.3	0.263
$(AV)_7$-A_{14}	AVAVAV	-28.3	0.047
V_{15}-A_{14}	VVVVVV	-29.4	0.294
$(A_3V)_3$-$(A_3V)_3$	AVAAAV	-26.5	0.017
$(A_3V)_3$-A_{14}	AVAAAV	-26.5	-0.076
$(AI)_7$-$(AI)_7$	AIAIAI	-29.1	0.317
A_{15}-$(AI)_7$	AIAIAI	-29.1	0.074
$(AIA_2)_3$-$(AIA_2)_3$	AIAAAI	-26.8	0.109
$(A_3I)_3$-$(A_3I)_3$	AIAAAI	-26.8	0.04
$(A_3I)_3$-A_{14}	AIAAAI	-26.8	-0.064
A_{15}-$(A_3I)_3$	AIAAAI	-26.8	-0.064
$(A_2I)_4$-A_{14}	AAIAAI	-27.1	-0.029
IA_6IA_6I-A_{14}	AAAIAA	-26.1	-0.064

Table S 3: Mechanical properties of spinnable constructs and their standard deviation:

	Strength (MPa)	Strain at break (%)	Toughness modulus (MJ/m^3)	Diameter (µm)	Young's modulus (MPa)
A_{15}-A_{14}	44.09 ± 19.64	47.96 ± 55.82	18.19 ± 20.34	13.40 ± 3.70	1685 ± 466
$(A_3T)_3$-$(A_3T)_3$	67.80 ± 30.58	8.31 ± 15.15	4.70 ± 12.34	19.41 ± 8.51	2183 ± 921
$(A_3V)_3$-$(A_3V)_3$	70.76 ± 24.37	3.26 ± 1.82	1.31 ± 1.07	17.12 ± 2.95	2786 ± 861
$(A_3V)_3$-A_{14}	64.51 ± 19.73	78.59 ± 59.89	49.58 ± 45.26	9.09 ± 2.68	3348 ± 1121
$(A_3I)_3$-A_{14}	131.63 ± 31.87	160.44 ±37.00	145.63 ± 42.18	4.16 ± 0.78	3501 ± 948
A_{15}-$(A_3I)_3$	78.78 ± 34.87	203.52 ±120.39	125.33 87.99	10.88 ± 2.59	3045 ± 964
$(A_2I)_4$-A_{14}	45.41 ± 9.73	84.51 ± 89.26	37.01 ± 40.61	12.11 ± 3.33	2463 ± 653
$(A_3I)_3$-A_{14}[a]	95.68 ± 39.53	150.76 ± 57.09	90.96 ± 45.19	6.13 ± 4.00	2854 ± 868

a) protein expressed in a bioreactor

Table S 4: Secondary structure content as determined by FTIR spectroscopy:

	A_{15}-A_{14}	$(A_3T)_3$-$(A_3T)_3$	$(A_3V)_3$-$(A_3V)_3$	$(A_3V)_3$-A_{14}	$(A_3I)_3$-A_{14}	A_{15}-$(A_3I)_3$	$(A_2I)_4$-A_{14}
α-helix/ random coil (%)	40.2	38.5	32.9	30.3	31.3	35.0	39.5
β- sheets (%)	40.1	40.0	40.7	44.7	43.2	47.0	32.3
Other (%)	19.7	21.5	26.4	25.0	25.5	18.0	28.3
